# Balancing Microalgae and Nitrifiers for Wastewater Treatment: Can Inorganic Carbon Limitation Cause an Environmental Threat?

**DOI:** 10.1021/acs.est.0c05264

**Published:** 2021-03-03

**Authors:** Francesca Casagli, Simone Rossi, Jean Philippe Steyer, Olivier Bernard, Elena Ficara

**Affiliations:** † Dipartimento di Ingegneria Civile e Ambientale (DICA), Politecnico di Milano, 32, Piazza L. da Vinci, 20133 Milan, Italy; ‡ INRAE, Univ Montpellier, LBE, 102 Avenue des étangs, 11100 Narbonne, France; § Institut National de Recherche en Informatique et en Automatique (INRIA), Biocore, Université Cote d’Azur, 2004, Route des Lucioles − BP 93, 06902 Sophia-Antipolis, France

**Keywords:** Microalgae-bacteria process modeling, wastewater remediation, long-term validation, alkalinity, greenhouse gas emissions

## Abstract

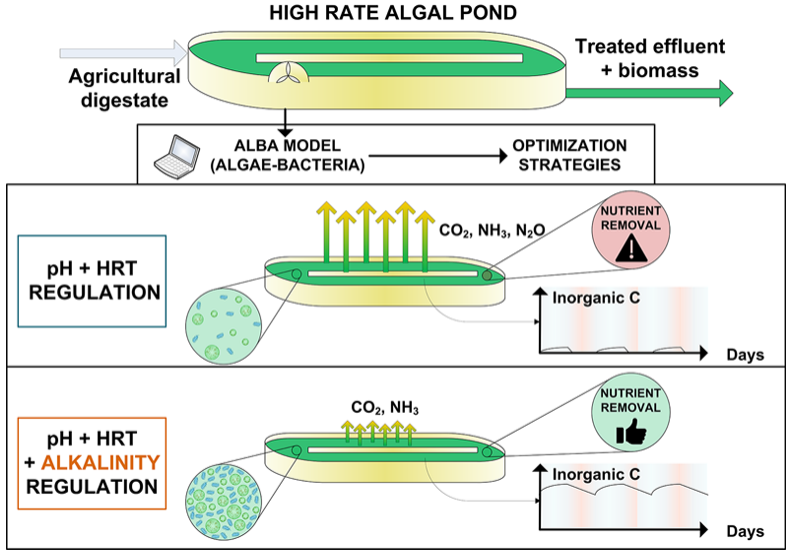

The first objective of this study is to assess the predictive capability of the ALBA (ALgae-BActeria) model for a pilot-scale (3.8 m^2^) high-rate algae-bacteria pond treating agricultural digestate. The model, previously calibrated and validated on a one-year data set from a demonstrative-scale raceway (56 m^2^), successfully predicted data from a six-month monitoring campaign with a different wastewater (urban wastewater) under different climatic conditions. Without changing any parameter value from the previous calibration, the model accurately predicted both online monitored variables (dissolved oxygen, pH, temperature) and off-line measurements (nitrogen compounds, algal biomass, total and volatile suspended solids, chemical oxygen demand). Supported by the universal character of the model, different scenarios under variable weather conditions were tested, to investigate the effect of key operating parameters (hydraulic retention time, pH regulation, k_L_a) on algae biomass productivity and nutrient removal efficiency. Surprisingly, despite pH regulation, a strong limitation for inorganic carbon was found to hinder the process efficiency and to generate conditions that are favorable for N_2_O emission. The standard operating parameters have a limited effect on this limitation, and alkalinity turns out to be the main driver of inorganic carbon availability. This investigation offers new insights in algae-bacteria processes and paves the way for the identification of optimal operational strategies.

## 1 Introduction

High Rate Alga-Bacterial Pond (HRABP) is a promising technology for wastewater treatment.^1,2^ The process overcomes some critical aspects of conventional biological processes by reducing the oxygen demand and opening new routes for nitrogen and phosphorus recovery. Indeed, the photosynthetic activity of microalgae can provide the necessary oxygen to support bacterial needs and therefore avoid the energy consumption associated with external aeration.^3−6^

In addition, combining removal of nitrogen and phosphorus by algae and nitrifying bacteria can enhance the nitrogen conversion capacity of the system. By providing oxygen to nitrifiers,^7^ algae substantially increase the overall ammonium removal capacity of the system,^8^ while nitrifiers reduce the oxygen level below the inhibition thresholds for algae.^9^ Nitrification, in turn, helps by keeping the ammonium concentration low, thus reducing the risk of free ammonia inhibition on algae, especially when high strength wastewaters are to be treated.^10,11^ Nitrifiers convert ammonium into nitrate that can be uptaken by the algae. The combination between algae and nitrifying bacteria has also some drawbacks, inducing negative interactions like the competition for CO_2_ or micronutrients^12^ or the inhibition of bacterial growth when the photosynthesis increases the pH level.^13^ These complex interplays make the overall dynamics of nitrifiers/algae especially challenging to understand and predict, as well as highly dependent on the composition of the wastewater to be treated and on the operation parameters.^14^ The HRABP efficiency is also seasonal-dependent, and low temperature and solar radiation conditions can seriously affect the overall microalgae growth and its synergy with bacteria,^15^ potentially leading to the collapse of the system.^16^ Moreover, the overall dynamics of the algae/bacteria community can affect atmospheric emissions, not only in terms of free ammonia stripping but also by modulating the conditions promoting N_2_O emission.^17−19^

These inherent complexities explain why contrasting conclusions have been drawn on the synergy and competition between algae and bacteria.

Mathematical models are powerful tools for understanding, predicting, and optimizing bioprocesses, especially for unraveling the complex nonlinear interactions among microorganisms. As for anaerobic digestion and activated sludge processes, reference validated models ADM1^20^ and ASMs^21^ turned out to be efficient tools currently used for supervision and process operation. To date, more than 300 models are available focusing on microalgae metabolism.^22^ However, only a minority of them were developed for modeling mixed algal-bacterial cultures in raceway ponds. Most of them were not calibrated and/or validated on a long-term data set (i.e., beyond 1 week of experimental data in outdoor conditions). In addition, only nine models for wastewater remediation adopted the IWA standard nomenclature with an explicit stoichiometric matrix, thus facilitating their integration with plant-wide modeling platforms.

The ALBA model^23^ integrates all the physical (temperature, evaporation, gas–liquid exchange, hydraulics), chemical (acid/base equilibria and pH), and biological (algal and bacterial growth and decay) submodels required to efficiently predict the system behavior. The ALBA model was first presented in Casagli et al.^23^ The model was built up to simulate algal-bacterial synergistic interactions and competitions, to evaluate the HRABP remediation performances, and to explore the feasibility of integrating it in existing WasteWater Treatment Plants (WWTPs) to convert them into Water Resource Recovery Facilities (WRRFs).

The biological dynamics of the ALBA model is driven by the functions representing the influence of environmental factors (light, temperature, pH, oxygen) and of nutrient availability through the Liebig’s minimum law. The ALBA model can be compared with existing reference models for bacteria (ASMs) and for algae-bacteria consortia,^24−29^ as reported in Table 1. For most of the models, the dependence of biomass growth on nutrient availability is modeled by the typical Monod-like kinetics. Only the modified ASM3 includes a version implementing phototrophic growth on nitrogen storage compounds. Other published models on algae metabolism adopt the Droop model, such as the ASM_A,^30^ though they do not include interactions with bacteria and are therefore not reported in Table 1. Among the models in Table 1, only the modified RWQM1, the BIO_ALGAE2 and the ALBA models account for multiple nutrient limitations (nitrogen, phosphorus, and inorganic carbon).

**Table 1:** Comparison among the Main Algae-Bacteria Models Available in the Literature for Wastewater Remediation^a^

	RWQM1	PHOBIA	modified RWQM1^b^	modified ASM3	Bioalgae1	Bioalgae2	ALBA^b^
Reference	Reichert, 2001	Wolf, 2007	Broekhuizen 2012	Arashiro, 2017	Solimeno, 2017	Solimeno, 2019	Casagli, 2021/this work
Model Structure/Characteristics							
State variable (no.)	24	16	24	16	19	19	17
Biological processes (no.)	22	13	22	21	18	18	19
Parameters (no.)	120	75	138	47	94	108	72^c^
Growth kinetic type	multiplicative	minimum	multiplicative	multiplicative	multiplicative	multiplicative	multiplicative/minimum^d^
Dependence on organic and inorganic carbon	C_ORG_	C_ORG_, CO_2_, HCO_3_	C_ORG_, CO_2_, HCO_3_, CO_3_^2–^	C_ORG_	C_ORG_, CO_2_, HCO_3_	C_ORG_, CO_2_, HCO_3_	C_ORG_, CO_2_, HCO_3_, CO_3_^2–^
Considered N-forms	NH_3_, NH_4_^+^, NO_3_^–^, NO_2_^–^	NH_3_^+^, NO_3_^–^	NH_3_, NH_4_^+^, NO_3_^–^, NO_2_^–^, N_2_	NH_4_^+^, NO_3_^–^, NO_2_^–^	NH_3_, NH_4_^+^, NO_3_^–^, NO_2_^–^	NH_3_, NH_4_^+^, NO_3_^–^, NO_2_^–^	N_org_, NH_3_, NH_4_^+^, NO_3_^–^, NO_2_^–^ HNO_2_, HNO_3_, N_2_
Considered P-forms	H_2_PO_4_^–^, HPO_4_^2–^	-	H_2_PO_4_^–^, HPO_4_^2–^	-	SPO4^e^	SPO4^f^	H_3_PO_4_, H_2_PO_4_^–^, HPO_4_^2–^, PO_4_^3–^
Continuity check (mass conservation)	C, O, N, P	n.s.	C, O, N, P	(COD, N, P)	n.s.	n.s.	C, H, O, N, P, COD
Algal biomass composition	C_100_H_232_O_26_N_14_P	n.s.	C_100_H_232_O_26_N_14_P	C_106_H_181_O_45_N_16_P	C_100_H_232_O_26_N_14_P	C_100_H_232_O_26_N_14_P	C_100_H_183_O_48_N_11_P
Bacterial biomass composition	C_150_H_335_O_13_N_30_P	n.s.	C_150_H_335_O_13_N_30_P	C_5_H_7_O_2_N	C_150_H_335_O_13_N_30_P	C_150_H_335_O_13_N_30_P	C_60_H_87_O_23_N_12_P
PAR model	Steele	Eilers and Peters	Smith	Poisson	Eilers and Peters	Eilers and Peters	Bernard and Remond
pH model	NH_4_^+^, NH_3_, CO_2_, HCO_3_, CO_3_^2–^, H_2_PO_4_^–^, HPO_4_^2–^, Ca^2+^_,_ H^+^_,_ OH^–^	NH_4_^+^, NH_3_, CO_2_, HCO_3_, CO_3_^2–^, H^+^_,_ OH^–^_,_ Δ_CAT,AN_	NH_4_^+^, NH_3_, CO_2_, HCO_3_, CO_3_^2–^, H_2_PO_4_^–^, HPO_4_^2–^, Ca^2+^_,_ H^+^_,_ OH^–^	-	NH_4_^+^, NH_3_, CO_2_, HCO_3_ CO_3_^2–^, H^+^_,_ OH^–^	NH_4_^+^, NH_3_, CO_2_, HCO_3_, CO_3_^2–^, H^+^_,_ OH^–^	NH_4_^+^, NH_3_, CO_2_, HCO_3_, CO_3_^2–^, H_3,_PO_4_, H_2_PO_4_^–^, HPO_4_^2–^, PO_4_^3–^, NO_2_^–^, HNO_2_, NO_3_^–^, HNO_3_, H^+^, OH^–^, Δ_CAT,AN_, TA
pH growth dependence	-	-	Gaussian law	-	-	CPMI	CPM
Temperature simulation/growth dependence	-/Arrhenius	-	-/Arrhenius	-	-/Arrhenius	-/CTMI	√/CTMI
Ammonification	-	-	-	-	-	-	√
DO inhibition	-	-	-	-	√	√	√
NH_3_ inhibition	-	-	-	-	-	-	√
Gas–liquid mass transfer	O_2_	-	O_2_	-	O_2_, CO_2_, NH_3_	O_2_, CO_2_, NH_3_	O_2_, CO_2_, NH_3_, evaporation
Experimental Setup							
Reactor type	river environment	laboratory incubator	raceway	cylindrical photobioreactor	raceway	cylindrical photobioreactor	raceway
Reactor installation/volume	outdoor	indoor(lab)/3L	outdoor/8 m^3^	indoor(lab)/2 L	outdoor/1 m^3^	indoor(lab)/4 L	outdoor/17 m^3^, 1 m^3^
Influent	wastewater discharge	MM	MWW	DSC	MWW	MWW	SWW, DSC
Calibration data set	-	-	365 d	√ (24 h)	√ (4 d)	√ (8 d)	√ (30 d)
Validation							
Short-term dynamics	-	-		√ (24 h)	√ (4 d)	n.s.	3, 14 d
Long-term dynamics	-	-	330 d	-	√ (175 d)	-	√ (413 d, 189 d)
Sensitivity analysis	√	√	-	√	√	-	√
Seasonal analysis	-	-	-	-	-	-	√
Parameter uncertainty	√	-	-	√	-	-	√
Confidence intervals for model predictions	√	-	-	-	-	-	√

a Abbreviations: √: implemented; n.s. not specified or provided in the relative publications; IC: Inorganic Carbon; DSC: Diluted Swine Centrate; MM: Mineral Medium; MWW: Municipal WasteWater; SWW: Synthetic Municipal WasteWater; TA: Total Alkalinity; CTMI: Cardinal Temperature Model with Inflection; CPMI: Cardinal pH Model with Inflection; CPM: Cardinal pH Model.

b Demonstrative scale reactors (>5 m^3^).

c Not including chemical constants, their temperature dependence, and stoichiometric coefficients.

d In the ALBA model, only the Monod limitation terms relative to nutrients availability were implemented in the minimum function, while the dependence on inhibitory and environmental factors is multiplied for the minimum term (in the PHOBIA model, all the multiplicative terms considered are included in the minimum function).

e P limitation term only on algae.

f P limitation term on algae and bacteria.

To be accurate, models of outdoor processes must be proven successful in predicting the behavior over all four seasons and daily dynamics, induced by the solar and meteorological cycles for the fast-changing variables, such as dissolved oxygen (DO) and pH. The ALBA model was validated over a period of 413 days covering all the seasons both for daily and seasonal dynamics. Furthermore, none of these models are fully predictive because the temperature of the pond needs to be measured.

In this paper, the ALBA model–previously validated on a synthetic urban wastewater^23^ −was tested on a pilot-scale HRABP processing an agricultural digestate and located in a piggery farm in Northern Italy. The first objective was to challenge the ALBA model and its current calibration on a long time scale, including the start-up phase, with a different type of influent and different environmental conditions (*Csb* instead of *Csa* climatic area, according to the climatic classification proposed by Peel et al.^31^). Moreover, a simple but accurate sub-model was included to allow the ALBA model to forecast the in-pond temperature from air temperature data, making it a locally predictive tool to assess seasons or climate effects on a yearly time frame.

The second objective of this work is to optimize algal productivity and nutrient removal rates, while considering environmental impacts including atmospheric emissions. Specifically, the effect of key operation parameters such as hydraulic retention time (HRT), pH control set-point, and volumetric liquid/gas mass transfer coefficient (k_L_a) were considered. Simulations revealed the competition for inorganic carbon between nitrifiers and microalgae which not only degrades the system performance but also triggers conditions favorable for N_2_O production.

## 2 Materials and Methods

### 2.1 Case Study and Experimental Data Set

A detailed description of the experimental setup, field data collection, level of experimental replication, and treatment performances can be found in Pizzera et al.^32^ The HRABP, with a surface of 3.8 m^2^ and an operational volume of 0.88 m^3^, was installed on a large-scale piggery farm located in Northern Italy. In this farm, the excess sludge produced by the local WWTP was codigested with other agricultural wastes (chopped corn and barley, poultry manure, and olive pomace) in a biogas plant. The liquid fraction of the digested sludge (centrate) was separated by centrifugation and fed to the HRABP, after dilution with tap water to reduce nutrient concentrations. The HRABP was operated continuously for 189 days (31/05/2016–06/12/2016). Different dilution factors were applied to the centrate (i.e., a dilution factor of 5, until 17/09/2016, and a dilution factor of 3 until the end of the experimentation). The inflow rate was set to achieve an average HRT of 10 days until 11/10/2016; then, the HRT was increased to 20 days to compensate for temperature reduction. The mixing was ensured by a paddle wheel, operated at 20 rpm. The volumetric mass transfer coefficient (k_L_a) of the HRABP was experimentally determined during abiotic tests, resulting in a final value of 30.5 d^–1^ (as detailed in SI.12) which is very close to 34 d^–1^ estimated for the 56-m^2^ pond on the basis of which the model was calibrated.^23^ The reactor was equipped with a contact cylinder for CO_2_ bubbling to reduce pH when it overpassed a threshold (pH_threshold_ = 8 until 16/06/2016, then pH_threshold_ = 7.5). Influent characteristics were monitored once a week by measuring the organic matter content (total and soluble COD), inorganic nitrogen compounds (Total Ammoniacal Nitrogen, TAN and nitrate, N-NO_3_^–^), Total Kjeldahl Nitrogen (TKN), phosphate (P-PO_4_^3–^), and Total Suspended Solids (TSS). The mixed liquor in the HRABP was monitored twice a week, and the analyses were performed by spectrophotometric test kits on the following: TAN, nitrite (N-NO_2_^–^), nitrate, phosphate, and COD (total and soluble fractions). Optical density at 680 nm (to provide an estimate of the chlorophyll-*a* content), TSS, and volatile suspended solids (VSS) were measured once a week, together with algal cell counts. The reactor was also equipped with two online probes recording pH, temperature, and DO concentration (refer to Pizzera et al.^32^ for more details).

Standard deviations for on-line probes and off-line measurements were computed through the variation coefficient (further detailed in SI.2.).

### 2.2 ALBA Model Overview

All details on the ALBA model can be found in Casagli et al.^23^ and are recalled in SI.11. The biological sub-model (17 variables, 19 processes) considers a mixed culture of algae, heterotrophic bacteria, and nitrifiers, the latter including Ammonium Oxidizing Bacteria (AOB) and Nitrite Oxidizing Bacteria (NOB). The mass conversions are comprehensively described by the Petersen matrix, through which the conservation of COD, C, N, O, P, and H is verified. The photosynthesis response to irradiance is described by a reparametrized Haldane function and accounts for light penetration through the Beer–Lambert equation.^33^ A minimum law is used to describe the growth limitation from multiple substrates (carbon, nitrogen, and phosphorus). The pH sub-model is based on dissociation equilibria and ionic species mass balances, according to the one proposed in the ADM1 model.^20^ The CO_2_, NH_3_, and O_2_ stripping/dissolution were included, quantifying their rates through the k_L_a. The influence of pH on algae and bacteria metabolism is implemented using the function proposed by Rosso et al.,^34^ i.e., the Cardinal pH Model (CPM). The temperature dependence for growth and respiration rates is considered for all the biomasses and modeled by the Cardinal Temperature Model with Inflection (CTMI),^35^ while the Arrhenius function is chosen for modeling decay rates (see Table SI.11.6).

#### 2.2.1 Pond Temperature Submodel and pH Regulation Scheme

In order to simulate the process behavior under any meteorological condition, a simple model was developed to predict the temperature in the raceway.

This model estimates the in-pond temperature from the available air temperature data, and it was calibrated on this case study. More specifically, the pond temperature was calculated according to the following regression (eq 1, *R*^2^ = 0.98)
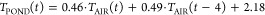
1where *T*_POND_(*t*) [°C] is the calculated pond temperature at time *t*, *T*_AIR_(*t*) is the measured air temperature at time *t*, and *T*_AIR_(*t*–4) is the air temperature measured 4 h before time *t*. The 4 h delay turned out to be optimal to best predict the pond temperature, see details in SI.7. Although more mechanistic models exist,^36,37^ they were not considered in this study. Unlike typical full-scale raceway ponds, the pilot-scale raceway was not lying on the ground (no conductivity with the ground). Developing a thermal model for this reactor would have required dedicated developments though without upscaling perspectives.

On top of the ALBA model, a pH control system simulated the operation conditions applied in the pilot plant under study. The pH control scheme was based on the injection of a pure CO_2_ flow when the pH value exceeded the set-point. The CO_2_ flow rate was proportional to the difference between the pH value and the pH set-point (SI.8).

#### 2.2.2 Numerical Integration

The software platform used for numerical simulations is AQUASIM,^38^ which has been used by many authors for modeling biological wastewater-treating systems.^25,27,39^ The raceway was simulated as a completely mixed reactor. Indeed, the mixing of the smaller raceways used in this study turned out to be close to ideal. However, large-scale ponds can be imperfectly mixed, and a more complicated hydrodynamics model must then be used (e.g., Demory et al.^40^).

#### 2.2.3 Model Validation and Evaluation Criteria

The ALBA model was previously calibrated and validated for 443 days from a demonstrative-scale raceway (56 m^2^) in France, treating synthetic municipal wastewater.^23^ More details regarding the calibration strategy can be found in our previous work.^23^ Briefly, a sensitivity analysis was first conducted by running simulations under typical seasonal conditions and following a periodic regime for the most relevant environmental conditions (i.e., light, temperature, and evaporation rate). This allowed the identification of a subset of most sensitive parameters, that were subsequently ranked according to the value of the absolute-relative sensitivity functions. A similar sensitivity analysis was performed here and provided similar results.

These parameters were then calibrated in Casagli et al.^23^ and were not modified here, even though the two case studies differ from the process design, wastewater type, and climate.

The parameter error propagation was computed using the sensitivity functions (see SI.13), and 95% confidence intervals on the modeled predictions were assessed.

Model performances were evaluated with the modified Theil’s Inequality Coefficient (TIC, eq 2)^41^
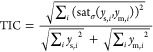
2where *y*_s,*i*_ is the simulated value at the time-point *i*; *y*_m,*i*_ is the measured value; and sat_σ_(*y*_s,*i*_,*y*_m,*i*_) is a function assuming a value of zero, when both *y*_m,*i*_ and *y*_s,*i*_ are lower than the standard deviation of the associated measurements (identifying an ideal fit) and assuming the value sat_σ_(*y*_s,*i*_,*y*_m,*i*_) = *y*_s,*i*_ – *y*_m,*i*_ otherwise.

The TIC value was computed for the whole set of experimentally observed variables (i.e., pH, DO, ammonium, nitrite and nitrate nitrogen, TSS, soluble COD, and algal biomass), either for the entire experimental campaign or season by season. The difference between simulated and observed values is normalized according to the amplitude of the variable, and the model is considered to have a good fit when the efficiency criterion approaches zero.

### 2.3 Meteorological Data and Climatic and Operational Scenarios

Meteorological data–including incident irradiance, air temperature, air humidity, wind speed, and rainfall–were provided by the Lombardy Environmental Protection Agency (ARPA Lombardia, www.arpalombardia.it). The evaporation rate was also investigated and computed on the basis of the available meteorological data (see SI.4), through the model provided by Béchet et al.^36^ A good prediction of the evaporation contribution is indeed fundamental because it significantly influences the hydraulic balance, hence the in-pond concentrations, further affecting light penetration and process rates.^42^

**Figure 1:**
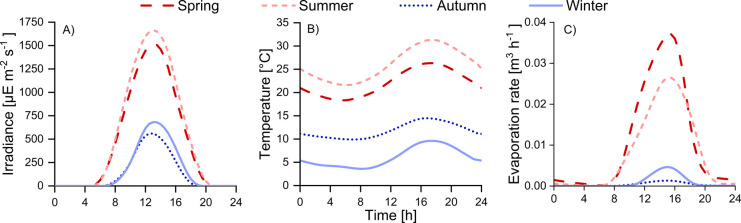
Average daily variation for each seasonal scenario: light (A), temperature (B), and evaporation rate (C).

Four different climatic scenarios representative of each season were developed computing the most relevant environmental conditions (i.e., light, pond temperature, and evaporation rate), by averaging hourly weather data over each season. In this way, a typical daily pattern was defined and extended to run simulations under established periodic regime (see Figure 1). The same average influent wastewater characteristics were considered for all seasons (Table 2). Subsequently, several operational scenarios were tested by varying the HRT, pH set point, k_L_a, and total alkalinity (TA), as reported in Table 3. Two extreme k_L_a values were selected, consistently with Casagli et al.^23^ The first value, k_L_a = 34 d^–1^, is typical of a condition of strong mixing in pilot-scale ponds, in which the algal-bacterial suspension is agitated through the paddle wheel and a high mass transfer rate is obtained. This high k_L_a value was estimated for the 56-m^2^ pond used to calibrate the ALBA model,^23^ in line with other studies at similar scale.^43−45^ The second value, k_L_a = 0.5 d^–1^, represents a condition of reduced mass transfer. This lower k_L_a value represents poor agitation with limited atmospheric gas exchange that is typical of either full-scale ponds or raceway reactors provided with alternative mixing systems (such as submerged propellers), that guarantee appropriate mixing while minimizing the gas transfer.^46^ This lower value has also been recorded for raceway channels and shallow ponds.^47−49^

**Table 2:** Summary of the Measurements Taken during the Monitoring Campaign: Influent Characteristics, Online Reactor Probes, and Environmental Conditions

Influent characteristics								
	COD_T_	CODs	TAN	N-NO_3_^–^	P-PO_4_^3–^	TAN/TKN	TSS	Turbidity
Unit	mgCOD L^–1^	mgCOD L^–1^	mgN L^–1^	mgN L^–1^	mgP L^–1^	mgN mgP^–1^	mgTSS L^–1^	FAU
Value (mean ± st.dev.)	514 ± 190	381 ± 114	310 ± 91	12 ± 5	14 ± 4	0.85 ± 0.1	146 ± 0.1	127 ± 145
Frequency	once a week							

In-reactor probes			
Parameter	Value		
Probe	O_2_	pH	T
Unit	mg L^–1^	-	°C
Value (mean ± st.dev.)	8.6 ± 3.3	7.1 ± 0.5	20.9 ± 7.7

Environmental conditions (maximum; average)			
	Spring	Summer	Autumn
Incident irradiance [μmol m^–2^ s^–1^]	1572; 470	1742; 529	825; 196
Source	ARPA Lombardia meteo data set		

**Table 3:** Parameters Set for the Selected Operational Scenarios

Parameter tested	Scenario no.	HRT (d)	pH set-point^a^	k_L_a (d^–1^)
k_L_a	S1	10	7.5	34
	S2			0.5
HRT	S3	2	7.5	34
	S4	5		
	S5	15		
	S6	20		
pH	S7	5	6.5	34
	S8		7	
	S9		8	
	S10		NC^c^	
TA^b^	S11	5	7.5	34

a The pH control system was implemented in the model as reported in SI.8, simulating one where the maximum pH value set was regulated with pure CO_2_ injection.

b In this scenario, the concentration of TA (expressed in mol m^–3^) in the influent was increased.

c NC: no pH control.

### 2.4 System Performance Criteria

The raceway performances were assessed based on the resulting algal biomass productivity and nutrient removal rates (ammoniacal nitrogen and orthophosphates) (Figure 7), computed as:
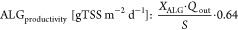
3
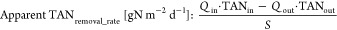
4
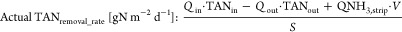
5
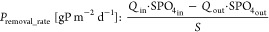
6where *X*_ALG_ is the algae concentration (gCOD m^–3^); 0.64 gTSS gCOD^–1^ is the conversion factor for algal biomass from COD to TSS computed from the algal stoichiometry; *Q*_in_ and *Q*_out_ (m^3^ d^–1^) are the inflow and outflow rates; TAN_in_ and TAN_out_ (gN m^–3^) are the ammoniacal nitrogen concentration entering and leaving the system; SPO_4in_ and SPO_4out_ (gP m^–3^) are the soluble P concentration as orthophosphate entering and leaving the system; QNH_3,strip_ (gN-NH_3_ m^–3^ d^–1^) is the ammonia transfer rate to the atmosphere (negative term representing the ammoniacal nitrogen fraction leaving the system through stripping, see Table SI.11.7); and *S* (m^2^) is the raceway surface. The apparent TAN removal rate (eq 4) was estimated by accounting for the influent and effluent TAN loads in the liquid only, while the actual TAN removal rate (eq 5) was computed by excluding the stripped N-NH_3_.

### 2.5 Alkalinity Computation

In order to clarify the role played by alkalinity, we computed the total alkalinity according to Dickson^50^ and Wolf-Gladow et al.^51^:
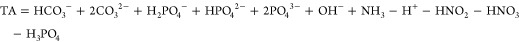
7An extension of this formula is given in SI.9, in order to account for a digestate which would contain volatile fatty acids (VFA) and hydrogen sulfide, which can be further used for a more general plant-wide model, coupling anaerobic digestion and HRABP models.

## 3 Results and Discussion

### 3.1 Long-Term Model Validation

**Figure 2:**
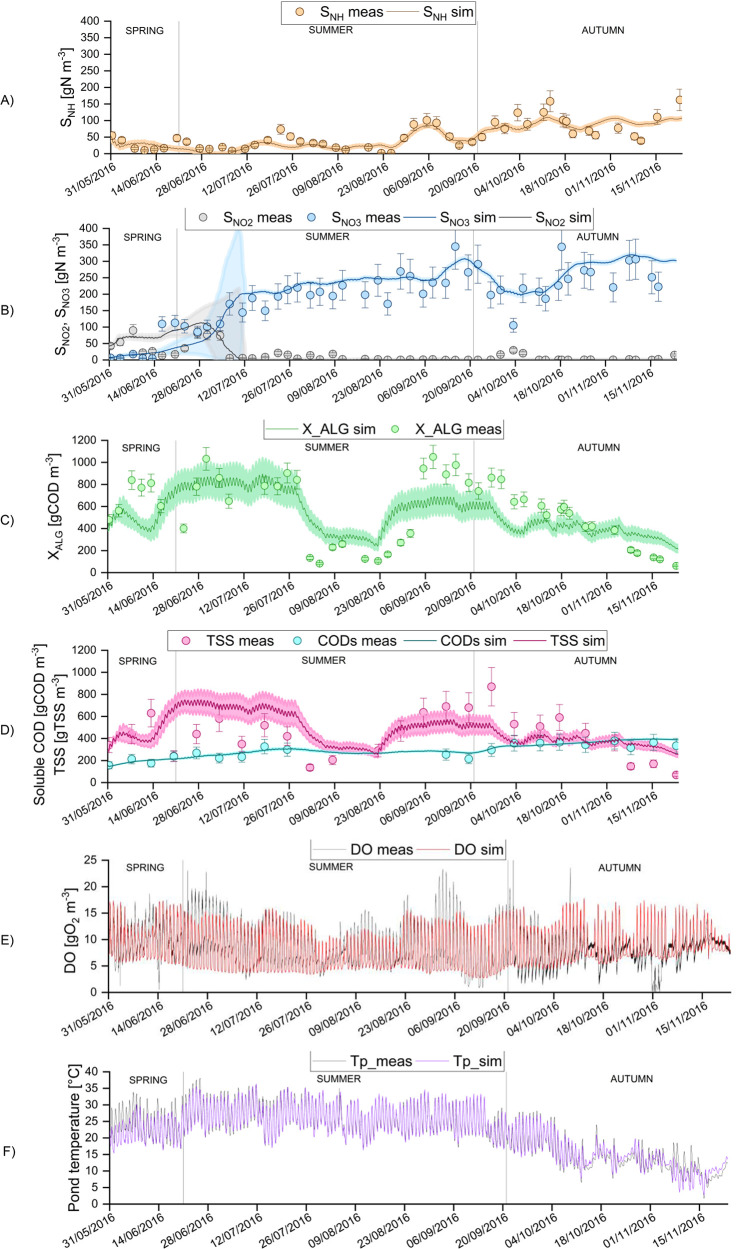
Long-term evolution of simulated (continuous line) versus measured values (dots): total ammoniacal nitrogen (A), nitrite and nitrate (B), algal concentration expressed in COD compared with measurements derived from optical density (C), soluble COD and TSS concentrations (D), DO (E), and temperature (F). Error bars on experimental measurements illustrate the standard deviations. Shaded areas on model predictions show the 95% confidence intervals.

The predictions of the ALBA model, and their 95% confidence intervals, were derived from the set of parameters previously calibrated^23^ and compared with the data from the six-month experimental campaign (Figure 2). The model performances were quantified using the TIC criterion (Table 4). The model quality score confirmed the good predicting ability of the model, considering that the model is said to be accurate for TIC values below 0.3.^41,52^ For all the tested seasons, experimental data were well simulated. Only the results obtained in spring for nitrogen forms were less accurate (Figure 2A and Figure 2B). This is due to the fact that experimental data in spring mainly belong to the start-up phase, during which nitrite tends to accumulate and simulations are affected by the selection of the initial conditions. The highest accuracy is obtained for the online measurements of pH, DO, and temperature, with a total TIC of 0.05, 0.15, and 0.09, respectively. This is probably the consequence of the calibration strategy based on these cornerstone variables.

**Table 4:** Model Efficiency Evaluated for Each Season

	Theil’s Inequality Coefficient – TIC			
	Total	Spring	Summer	Autumn
Temperature	0.09	0.09	0.10	0.10
DO	0.15	0.14	0.15	0.16
pH	0.05	0.04	0.04	0.08
S_NH_	0.20	0.31	0.21	0.20
S_NO2_	0.34	0.38	0.30	0.80
S_NO3_	0.10	0.55	0.08	0.10
X_ALG_	0.20	0.24	0.19	0.21
TSS	0.21	0.27	0.18	0.22
COD_S_	0.06	0.07	0.07	0.05

The nychthemeral oscillations of DO (Figure 2E) are generally well captured by the model, even if the predicted extreme values sometimes differ from the measurements.

In Figure 2C, the simulated algal biomass concentration is compared with the estimates derived from the optical density at 680 nm (see SI.3). The overall algal trend is well predicted by the ALBA model, as confirmed by the values of the TIC criterion (0.19–0.24). During the summer period, algal biomass strongly decreased. This was due to the setting up of a shading net in the period between 30/07/16 and 24/08/16, to reduce solar radiation and potential light inhibition. However, the shading-net effect was too strong and negatively affected algae growth, so that it was eventually removed. The predicted algae concentration responded markedly to these changes, and the predictions well fit the observed trend. In September, the model underestimates algal biomass, as confirmed by the higher TIC value. The observed misfit is probably partially due to the loose relationship between absorbance at 680 nm and algal biomass, which can vary due to photoacclimation, depending on light intensity, temperature, and nitrogen availability.^53^ Moreover, metagenomic analyses as well as microscopic observations (data not shown) evidenced sudden blooms of algal biomass predators (especially of *Vorticellae*), in particular between 20/6 and 30/6, which caused a significant reduction in the algal biomass concentration. Predators’ dynamics is not included in the ALBA model, and this could have caused local discrepancies.

Figure 2A and Figure 2B show the trend in nitrogen compounds. After about 20 days from the start-up, there was a switch from partial to total nitrification. Through the ALBA model, this remarkable event was effectively predicted. The same phenomenon was observed in the HRABP treating synthetic municipal wastewater that was previously used to calibrate and validate the ALBA model.^23^ Here, complete nitrification was reached only after 70 days from start-up. The very different context of these two algal-bacterial raceways (climate, operational strategies, influent characteristics, initial nitrifying biomass) explains the difference in the time horizon to achieve the complete oxidation of the ammoniacal nitrogen. Accurately simulating the dissolved inorganic nitrogen compounds is challenging, since these variables can be affected by almost all the processes taking place in the reactor. It is also worth noting that the decrease in algal concentration during midsummer because of the presence of the shading net (Figure 2C) did not significantly affect the ammonium removal and that nitrifiers remained mainly responsible for ammonium uptake. Indeed, in the same period, temperature and pH were close to the optimal values for nitrifiers growth. In addition, DO has never been a limiting factor, since algal photosynthesis, together with gas/liquid mass transfer, supplied enough oxygen to support the metabolism of nitrifiers.

Moreover, the predictions for the different dissolved inorganic nitrogen compounds are in acceptable ranges. From a practical perspective, the model has therefore a good predictive ability.

It should be highlighted that the TIC value for nitrite in autumn (0.80) is artificially high due to the very low measured and simulated values. Indeed, the TIC criterion is known to amplify small model misfits when values are close to zero.^54^ In fact, the model predicts values which significantly differ from the measurements only for five points out of 17. In the other seasons, TIC is always lower than 0.4. The simulated nitrite had a yearly average of 17.8 ± 33.8 g N m^–3^, while the measured nitrite was on average 14.7 ± 23.1 g N m^–3^.

This overall ability of the model to capture, without any recalibration, the system dynamics can be further appreciated in SI.14 (correlation between measurements and predictions, residuals analysis).

In summary, the model efficiently predicts both qualitatively and quantitatively the observations. This evidence demonstrates the model’s sound prediction capability.

### 3.2 Ecosystem Structure

A close look at the simulated dynamics of the involved microorganisms definitely confirms a structurally different system. To better identify the behavior of the system under consistently different conditions, the fractionation of the total biomass concentration was calculated by taking advantage of the capability of the ALBA model to predict the concentrations of the algal, heterotrophic, and nitrifying populations. Indeed, one of the most difficult aspects in mixed algae-bacteria systems is to experimentally determine the evolution of bacterial populations, since the quantification of the algal biomass is generally the only available measurement in the microbial community. Therefore, the percentage of each microbial guild on the overall biomass was computed. This result was compared with the microbial community composition simulated by the ALBA model.^23^ In the study reported in Casagli et al.,^23^ the HRABP was fed on synthetic urban wastewater, and microalgae were 76.8% of the biomass, heterotrophs were 21.8%, and nitrifiers were 1.4%, on average. In the current case study, the total biomass concentration was similar, but nonetheless, the composition was significantly different. Indeed, microalgae dominate (90.2%) the microbial community, while heterotrophs and nitrifiers are found in similar proportions (3.8% and 6.0% on average, respectively, see Figure SI.6.1A,B). Therefore, the ecosystem is definitely more autotrophic. Specifically, model simulations revealed that the AOB percentage on the total biomass was 4.9 ± 3.7%, and these predictions match the measurements (2.8 ± 1.7%) carried out by Mantovani et al.^6^ on a similar raceway located in the same area and processing digestate.

### 3.3 Model Universality for Significantly Different Conditions

Validating the model for a different case study (treating different wastewater with pH regulation and different climate) without modifying the set of parameters is definitely challenging and not at all straightforward.

The ALBA model could be better tailored to the current case study. For instance, some improvements could be achieved by tuning parameters affected either by the microbial composition (i.e., the light extinction coefficient) or by the process design (typically the k_L_a). Also, the parameters could be adapted to seasonal variability to track the changes in the microbial community evolution.

However, the quality of its performance does not motivate any further fine-tuning utilizing the data from the monitoring campaign, demonstrating the universality of the ALBA model and its parameters.

### 3.4 Short-Term Oxygen Dynamics

**Figure 3:**
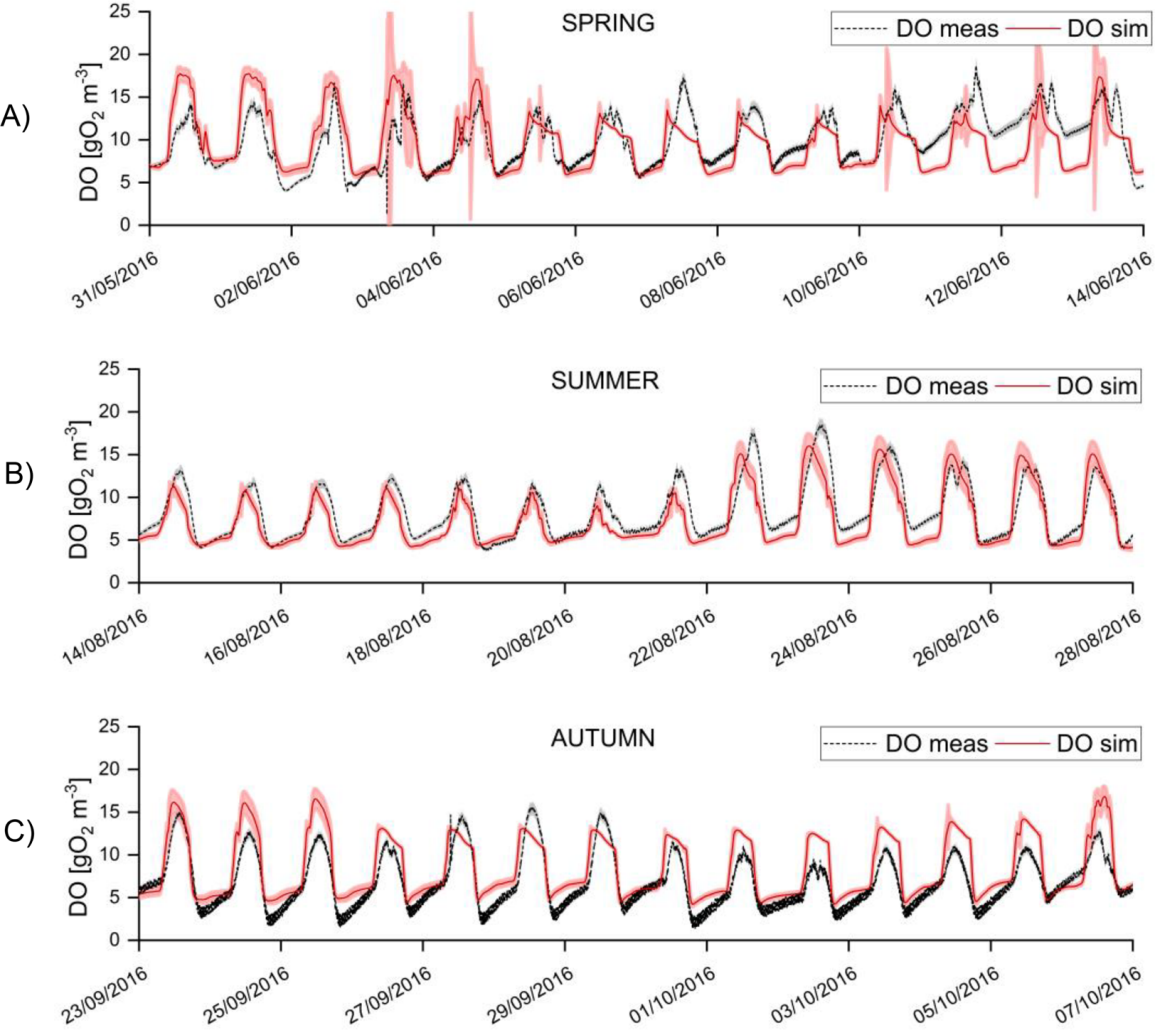
Short-term model validation: measured and simulated oxygen trend in spring (A), summer (B), and autumn (C). Gray shaded areas represent the standard deviation of DO online measurement. Red shaded areas represent the 95% confidence intervals of model predictions for DO.

In Figure 3, two selected weeks from the data set are shown in order to better appreciate the daily dynamics of DO in different seasons. In general terms, the model provided a very good agreement with experimental values. It can be observed that the model is able to follow the day/night cycles and that DO concentrations are effectively predicted during each season. This also occurs in the summer, when the shading net was applied.

The 95% confidence interval on model predictions is wider in the cloudy days in spring, as it is also confirmed by the recorded DO oscillations. This phenomenon highlights the sensitivity of the photosynthesis model to fast change in light intensity due to the clouds.

It is also interesting to notice that at night, when DO is only consumed by respiration, DO concentration increases, as observed in similar open systems.^23,55^ This apparently counterintuitive phenomenon, that the model was able to capture, is mainly due to the oxygen exchange with the atmosphere, that is enhanced at night by the increased oxygen solubility at lower temperatures. This results in a sufficient DO supply to support aerobic processes at night, i.e., the algal and bacterial respiration, which are, in turn, slowed down at lower temperatures. The occurrence of anoxic processes at night was avoided by the high reaeration rate provided by the paddle wheel (see Figure 2E), as it is generally the case with HRABPs.^2^ Indeed, at industrial scale (and lower k_L_a), lower oxygen concentration would be reached during the night.

### 3.5 Carbon, Nitrogen, and Oxygen Fluxes in the System

**Figure 4:**
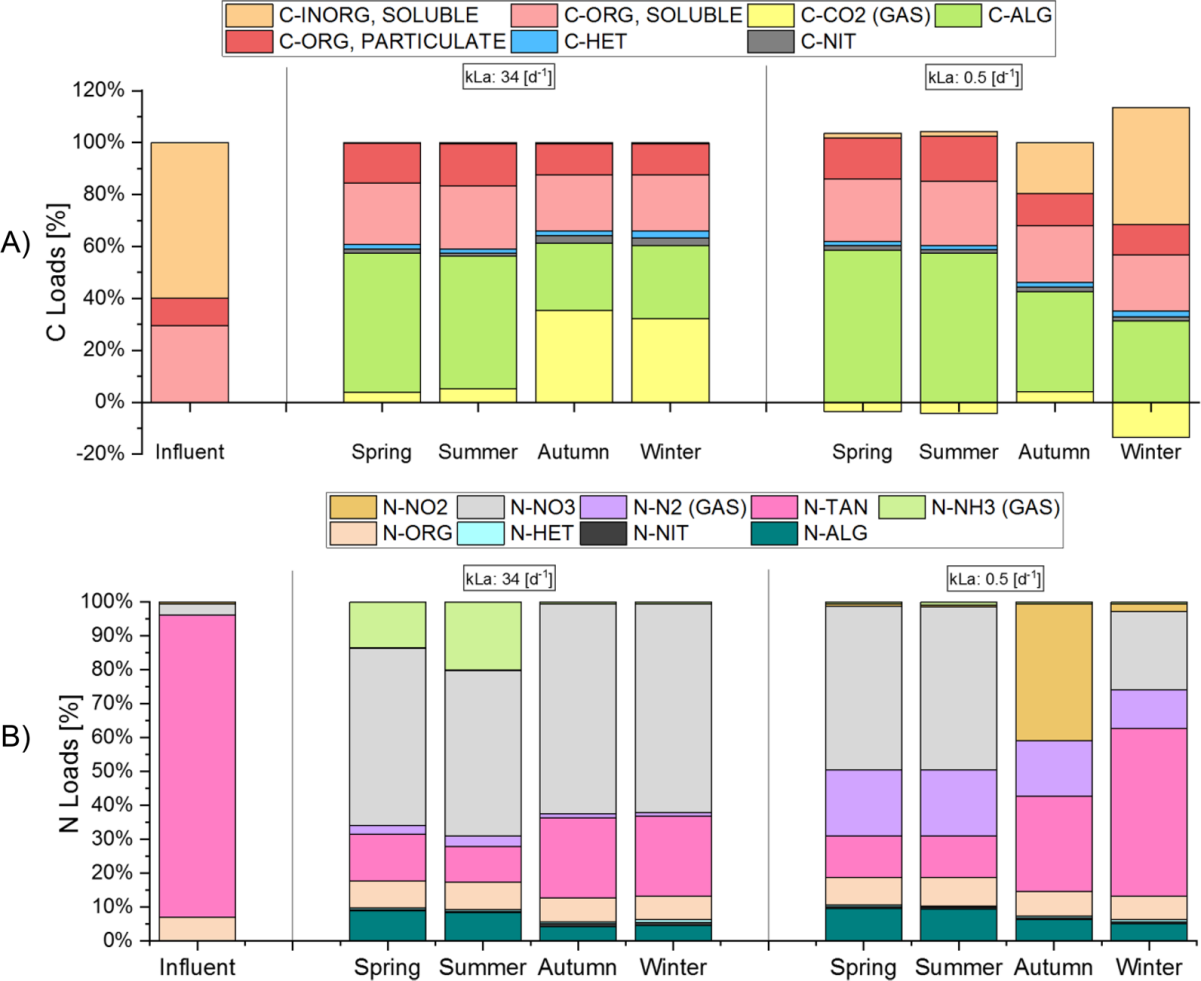
Percentage of influent and effluent carbon (A) and nitrogen (B) fluxes, under normal and reduced mass transfer conditions (S1, k_L_a = 34 d^–1^ and S2, k_L_a = 0.5 d^–1^). The S1 and S2 scenarios were analyzed according to seasons: spring, summer, autumn, and winter. In Figure 4A, C-ORG, PARTICULATE is the organic carbon present in *X*_S_ and *X*_I_ fractions; C-ORG, SOLUBLE is the organic carbon present in *S*_S_ and *S*_I_ fractions; C-ALG, C-NIT, and C-HET are the organic fractions present in the algal, nitrifying (AOB and NOB) and heterotrophic biomass, respectively. In Figure 4B, N-ORG is the organic nitrogen present in *X*_S_, *X*_I_, *S*_S_, and *S*_I_ fractions; N-ALG, N-NIT, and N-HET are the organic nitrogen fractions present in the algal, nitrifying (AOB and NOB) and heterotrophic biomass, respectively. The computed fluxes of N_2_, NH_3_ and CO_2_ are gaseous, while all other are liquid fluxes.

The validated ALBA model is a powerful tool to provide deep insights into the hidden mechanisms behind this complex dynamic system. A typical operating scenario was simulated (scenario S1, HRT = 10 d, pH set point = 7.5, k_L_a = 34 d^–1^) considering each seasonal condition. To better understand the role played by oxygen as exchange money between the various microorganisms in the ecosystem,^23^ an alternative system for mixing was simulated using a propeller,^46^ that would result into appropriate mixing but reduced gas exchanges with the atmosphere and eventually a lower mass-transfer coefficient (k_L_a = 0.5 d^–1^). The seasonal periodic regime (see section 2.3, Figure 1) was used to estimate the fluxes of carbon, nitrogen, and oxygen in the system. The partitioning of carbon and nitrogen among the different components in the inflow and outflow (expressed in percentage of the total liquid inflow) is reported in Figure 4. Figure 4A shows that, for this operating scenario, a large fraction of the carbon leaves the system as algal biomass, especially in spring and summer. The C fraction in the algal biomass is reduced in autumn and winter, as a consequence of the lower temperature and irradiance (Figure 1). The marked reduction in algal growth during these cold and low irradiance seasons leads to a decrease in the flux of CO_2_ uptake by photosynthesis. In spring and summer, a very low flow of CO_2_ is emitted, while CO_2_ emission accounts for 30% of the carbon entering the system in winter. The influent and effluent organic fractions are similar, since they are hardly affected by seasonal variability. Coming from an anaerobic digestion process, the majority of the organic C in the influent is inert (both for the soluble and particulate forms) and leaves the system without being chemically or biologically transformed.

In this alternative S2 scenario (Figure 4A), the main carbon outflow is still due to algal biomass, similar to the reference case. This outflow is slightly higher compared to the reference case and less CO_2_ is emitted, especially in the colder seasons, as the system becomes even a net CO_2_ consumer. While a CO_2_ fraction is stripped in the scenario S1 (k_L_a = 34 d^–1^), it remains in the bulk under low k_L_a, so that more inorganic carbon is available for algal growth during the day. In autumn and winter, a larger amount of dissolved inorganic carbon leaves the system because of the overall lower algal and nitrifying activities compared to warmer conditions. However, when considering total liquid and gaseous inorganic carbon outflows, the two scenarios are not so different. This could mean that gas exchange induced by the mixing system is not strongly affecting carbon conversion.

**Figure 5:**
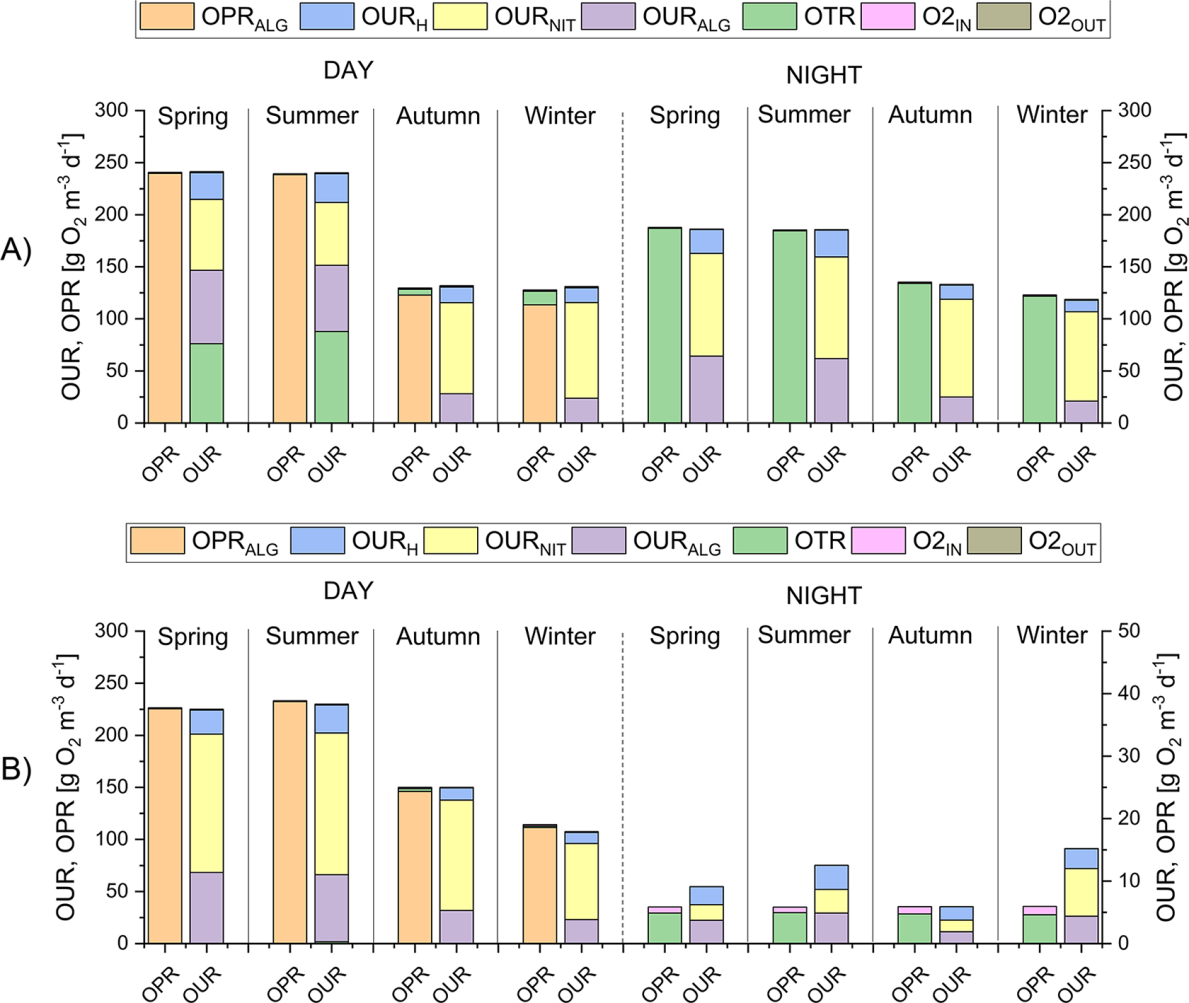
Oxygen production rate (OPR_ALG_), oxygen transfer rate (OTR), and oxygen consumption rates (OUR_ALG_, OUR_NIT_, OUR_H_) under two gas–liquid mass transfer conditions: A) scenario S1, k_L_a = 34 d^–1^ and B) scenario S2, k_L_a = 0.5 d^–1^. S1 and S2 scenarios were analyzed according to each season and day (left axis)–night (right axis) cycles.

The fluxes of oxygen in the system (separated into oxygen production rates, OPRs, and oxygen uptake rates, OURs) are given in Figure 5, distinguishing between day and night periods. Under the operating conditions S1 (Figure 5A), the oxygen production during the day is always sufficient to sustain the oxygen requirements due to the algal respiration and the bacterial activity, with a significant fraction of oxygen being wasted through stripping. The OPR is quite high in spring and summer, with a significant reduction in the cold seasons. The OUR of nitrifying bacteria is always higher than that of heterotrophic bacteria, coherently with the typically high TAN loads and the low levels of degradable carbon in digestates.^56,57^ The contribution of nitrifiers to the overall OUR is even larger in autumn and winter, further confirming that these microorganisms succeed in carrying out the TAN oxidation under cold conditions, also supported by the additional CO_2_ provided by the pH-control system. At night, the main oxygen input comes from liquid/gas transfer provided by the paddle wheel, thus supporting the algal and bacterial respiration.

With the S2 scenario simulating a mixing system with lower liquid/gas transfer (Figure 5B), the OPR is not particularly affected during the day, with values similar to the standard case. On the contrary, the nitrifying activity is highly enhanced in spring and summer, since more inorganic carbon and DO accumulate in the system, as they cannot be fastly transferred to the atmosphere. At night, however, the overall flow rates are reduced by at least one order of magnitude, while DO drops to zero for almost all the night because it is too slowly refueled from the atmosphere.

The different outflows of nitrogen are shown in Figure 4B. With paddle wheel standard operating conditions (S1), most of the nitrogen is oxidized to nitrate, evidencing the favorable conditions for the development of an algae-nitrifiers’ consortium. Nitrification is also active in cold seasons, thus contributing to maintain high TAN removal efficiencies all over the year. However, the unconverted TAN increases in winter, due to the reduced nitrifiers’ activity. Even if the nitrifying biomass is always lower than 10% of the algal biomass, the TAN fraction assimilated by the algae remains low all over the year, and approximately 56% of the TAN conversion route is via nitrification, on a yearly average. It should be noticed that, in spring and summer, the ammonia stripped from the pond can reach up to 20% of the influent load, while in the other seasons it is much less marked. This high NH_3_ emission has a strong environmental impact in terms of both eutrophication and greenhouse gas (GHG) emissions.^58^ This undesirable emission disappears when k_L_a decreases. In this case, a significant fraction (35–70% of the influent load) is still nitrified. However, due to a combination of temperature and DO limitation at night, that are unfavorable conditions for NOB, only a partial nitrification occurs in autumn, with nitrite being the largest fraction of the outflow nitrogen load. Algal activity is lower in autumn because of the reduced irradiance compared to winter (Figure 1). The larger outflow of molecular nitrogen at low k_L_a is due to the fact that DO drops to zero at night, making the anoxic growth of heterotrophic bacteria possible.

### 3.6 Can Algae and Nitrifiers Work Together without the Risk of N_2_O Production?

Avoiding inorganic carbon limitation is necessary for optimizing biomass productivity and nutrient removal rates.^59,60^ Working at pH below 7.5 is, in principle, well-known to guarantee that CO_2_ is not limiting the algal growth^59,61^ and nitrifiers’ activity.^62^ The model reveals that this statement does not hold here. Indeed, a closer look at alkalinity (SI.9) shows a regular drop caused by the consumption of ammonium as well as by the production of nitrate and nitrite. Influent alkalinity was 30 mol m^–3^, which is too low to support the full nitrification by autotrophic bacteria, as it has often been described for digestate.^63^ When the system reaches a very low alkalinity (0.40 ± 0.23 mol m^–3^, on average, according to eq 7), it no longer allows bicarbonate storage in solution, and the level of dissolved inorganic carbon remains dramatically low (see Figure SI.9.1). In all the simulated scenarios in which the pH was lower than 7.5 (and with HRTs that do not lead to biomass washout), the simulated concentration of inorganic carbon remained very low (below 8 gC m^–3^, see tables in SI.10) and resulted in the limited growth of autotrophic and photoautotrophic populations. Under these conditions, a strong competition for inorganic carbon takes place between the algae and the nitrifiers. The outcome of this competition, which also depends on other environmental (light and temperature) and chemical (DO, pH, and alkalinity) factors, contributes to defining the biomass distribution and the system dynamics.^64,65^ This analysis shows that inorganic carbon and alkalinity should be considered as key parameters to be controlled, especially in algae-bacteria systems, where the algal population dominates the system.

Conditions of inorganic carbon limitation have been shown by several authors to enhance cellular maintenance energy of nitrifiers and become favorable for the production of N_2_O.^66,67^ A wide range of microbial mechanisms has been identified as favoring N_2_O production in nitrifying systems, being chiefly dependent on environmental conditions and on the chemical characterization of the medium.^68,69^ N_2_O production by nitrifiers was studied by Mellbye et al.^67^ who observed a 6.3-fold increase when inorganic carbon became highly limiting. It is thus of utmost importance to identify the working conditions which are likely to give rise to a marked inorganic carbon limitation.

**Figure 6:**
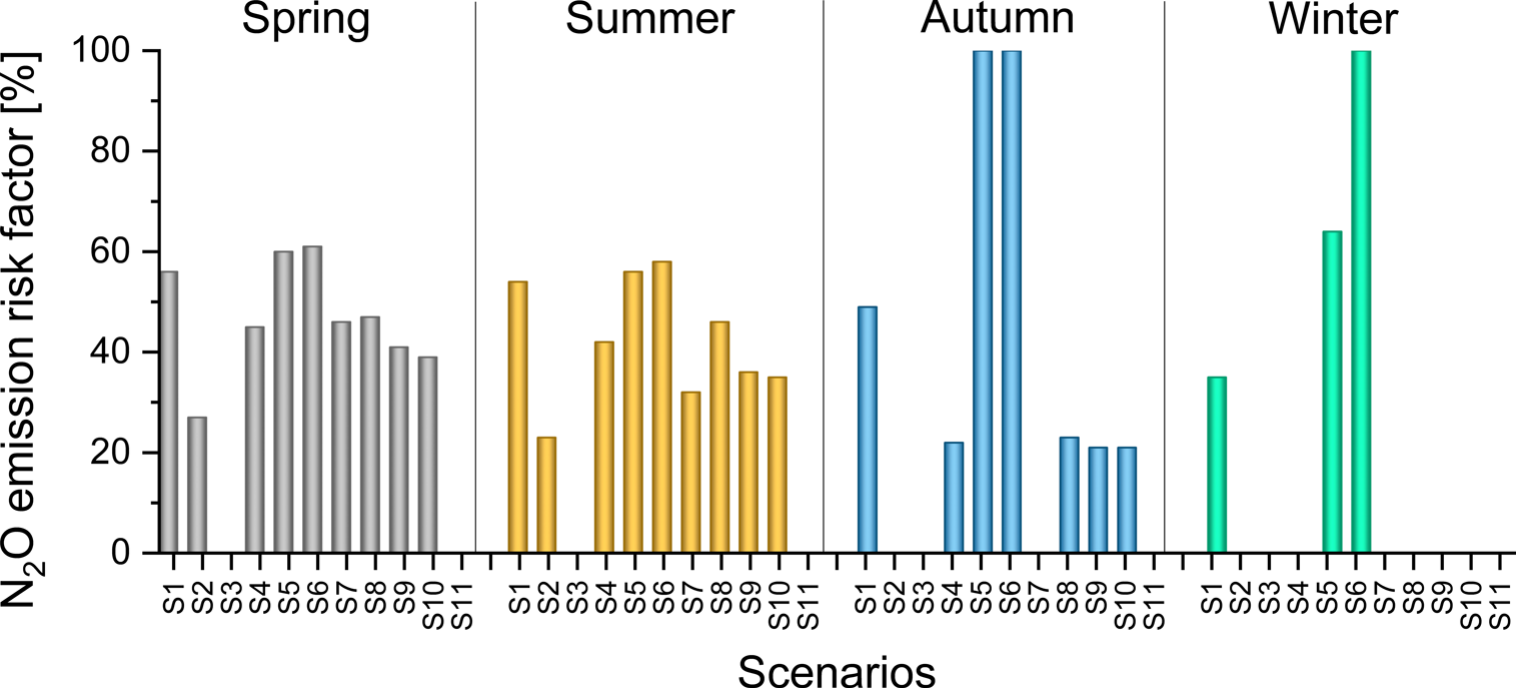
N_2_O emission risk factor (percentage of time along the day for which N_2_O formation conditions occur, i.e., inorganic carbon < 0.2 molC m^–3^), according to the season.

Although mechanistic models of N_2_O production already exist,^70^ further studies would be necessary to integrate the set of complex conditions triggering N_2_O emissions into a predictive model and to validate it under outdoor conditions. While developing a model able to represent the production of N_2_O and integrate it into the ALBA model is beyond the objectives of this work, the ALBA model can definitely help in identifying the critical working conditions associated with N_2_O production. The accurate modeling of the interplay between the chemical species driving the pH dynamics was used to evaluate the periods of strong inorganic carbon limitation, which have been shown to trigger N_2_O emissions, i.e., when the total inorganic carbon drops below 0.2 mol m^–3^, as suggested by Mellbye et al.^67^ The model therefore provides a risk index of N_2_O emissions (Figure 6), quantified as the percentage of time under which the system was likely to favor the bacterial N_2_O production. On the basis of this risk index quantification, the above investigated scenarios (S1 and S2) are associated with a high risk of N_2_O emission, especially in summer and spring, with more than 40% of the time operating under inorganic carbon limitation. The next paragraph investigates optimization strategies, so as to maximize the HRABP efficiency while avoiding operating under these dangerous conditions.

### 3.7 Can Raceway Performances Be Optimized Keeping Gaseous Emissions Low?

The ALBA model was used to explore the impact of different working conditions, simultaneously evaluating their effect on the gaseous emissions that are likely to have high environmental impacts (CO_2_, NH_3_, N_2_O) and on conventional efficiency parameters (algal productivity, nutrient removal). In a first stage, operating parameters classically used at industrial scale (HRT and pH-control set point) were considered.

#### 3.7.1 Classical Operating Management with HRT and pH

**Figure 7:**
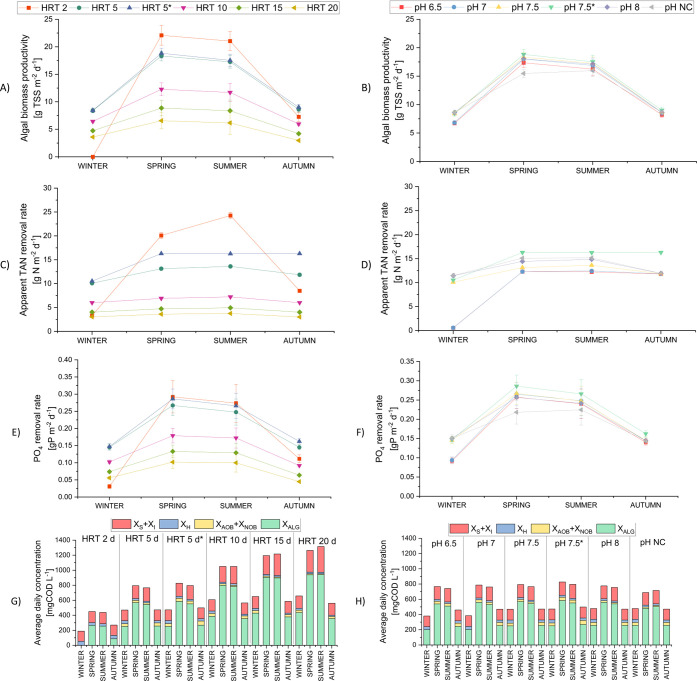
Effects of pH and HRT variation on the algae-bacteria cultivation in terms of the following: algal biomass productivity (A: HRT variation, B: pH variation), apparent TAN removal rate (C: HRT variation, D: pH variation), orthophosphate removal rate (E: HRT variation, F: pH variation), and TSS percentage fractionation (G: HRT variation, H: pH variation). X_S_ is the particulate slowly biodegradable organic matter, while X_I_ is the particulate inert organic matter. Simulations at different HRTs were run at a pH set point of 7.5 (scenarios S1, S3–S6, S11), while simulations at different pH set points were run with HRT = 5 d (scenarios S4, S7–S11).

Various scenarios were run exploring different combinations of HRT and pH (S3 to S10, Table 3), in order to show how these operational parameters can shape the ecosystem. Results are shown in Figure 7 (see all the tested conditions in SI.10). The algal biomass productivity (Figure 7A and Figure 7B) is strongly affected by the HRT. More specifically, by maintaining a 2-day HRT, productivity can reach values up to 22 and 21 gTSS m^–2^ d^–1^ in spring and summer, respectively. However, such a dilution rate leads to the washout of the algal biomass in winter, causing N and P removal rates to drastically drop (Figure 7C and Figure 7E). The best algal biomass productivity in autumn and winter is obtained for a 5-day HRT, with 8.5 gTSS m^–2^ d^–1^. Playing with pH resulted in a marginal effect only (Figure 7B), and it was used to further tune the optimal working modes.

It is worth noticing that the conditions optimizing algal productivity also maximize nutrient removal rates (Figure 7C and Figure 7E). For TAN, the apparent removal rate is in the range of 20 to 24 gN-NH_4_^+^ m^–2^ d^–1^ in spring and summer at HRT = 2 d, while it drops to 10–13 gN-NH_4_^+^ m^–2^ d^–1^ in autumn and winter at 5-day HRT. For phosphorus, it ranges from 0.24 to 0.29 gP-PO_4_^3–^ m^–2^ d^–1^ in spring and summer at 2-day HRT, while it drops from 0.1 to 0.15 gP-PO_4_^3–^ m^–2^ d^–1^ in autumn and winter, both for 2- and 5-day HRT.

Regulating pH at 7.5 seems a good trade-off to minimize the CO_2_ injection cost—even if CO_2_ can be recovered after biogas upgrading—while keeping a high algal productivity. In autumn and winter, pH 8 or even unregulated pH are appropriate, which will be the best solution from an economic point of view.

Atmospheric emissions must be considered in the optimization strategy to make sure that the process would be sustainable from an environmental point of view. The ALBA model was used to assess the flux of NH_3_ which is stripped to the atmosphere. In this view, a lower pH is highly recommended, especially in spring and summer when algal activity is higher, so that TAN mostly remains under the ammonium form and the undesirable flux of NH_3_ toward the atmosphere is strongly reduced (from 2–3 gN-NH_3_ m^–2^ d^–1^ at pH higher than 7.5 to 0–0.08 gN-NH_3_ m^–2^ d^–1^ at pH lower than 7.5, see SI.10). The actual nitrogen removal rate was also computed excluding stripping from the pond. This significantly changes the picture. For instance, looking closer at the fraction of the different populations in the system (see Figure 7G) for a low HRT, nitrifiers are washed out from the system. Ammonium is only consumed by algae at a much lower rate, and the actual TAN removal rate is finally very low. This is the reason why inorganic carbon was not limiting in these regimes, i.e., the low nitrification did not exert any pressure on it.

The way to consider the flux of CO_2_ emitted from the respiration processes of algae and bacteria is debatable since it is of biogenic origin. The emissions of the CO_2_ from the pH regulation system can be of fossil origin–if not recycled from the biogas, and more clearly contribute to greenhouse gas emissions. In any case, only an in-depth LCA study^71,72^ can accurately identify the process impact on climate change, but for sure it will be strongly dependent on the emission of gases such as NH_3_ and N_2_O. These emissions can be strongly reduced by correctly managing the HRABP, as it is discussed in the next section.

#### 3.7.2 Introducing Alkalinity in the Management Strategy

Finding a working mode balancing conventional efficiency parameters and atmospheric emissions is however challenging. The conditions maximizing both algal productivity and removal rates put pressure on the inorganic carbon stock, hence leading to the high risk of N_2_O production. The risk of N_2_O production appeared in all the scenarios (SI.10 and Figure 6), except for scenario S3, where nitrifiers are washed out of the system resulting in poor actual nitrogen removal. Spring and summer were the most affected seasons, and none of the classical operating conditions could avoid limitation by inorganic carbon for at least 30% of the time. In the scenarios where a low k_L_a was set (SI.2 and SI.4.1, also SI.10), the inorganic carbon limitation and the risk of N_2_O production were strongly reduced.

Subsequently, another scenario was considered and run under the same conditions set for scenario S4 but with influent alkalinity increased by 20 mol m^–3^ (named S11). The idea was to counterbalance the low alkalinity level responsible for the very low soluble inorganic carbon concentration despite pH regulation.

Under S11 conditions, the percentage of time for which N_2_O formation can occur drops to zero. In addition, in all the seasons, CO_2_ emissions are consistently lowered thanks to the higher capacity to store inorganic carbon in the system, as confirmed by the higher concentration of dissolved inorganic carbon (above 100 mol m^–3^, see SI.10). Simulations show that this scenario outcompetes most of the other scenarios and simultaneously maximizes the algal biomass production and nutrient removal. Moreover, the actual TAN removal rate increases since the inorganic carbon is no longer limiting as it was in S4. This finding shows that nitrifiers suffer from the competition for the available inorganic carbon under alkalinity-limiting conditions.

Alkalinity was revealed to be a hidden process parameter that must be definitely controlled to operate the system under optimal conditions and alkalinity addition is definitely a way to enhance the system performances.

An economic analysis with NaOH addition to regulate alkalinity was performed (see details of hypotheses and computations in SI.15). Treating an additional 30% of nitrogen (scenario S11 compared to S4), with additional alkalinity, has an estimated value of 0.03 $ m^–2^ d^–1^, computed on the basis of an operational cost for treating nitrogen of 6 $ kgN^–1^.^73^ The cost of alkalinity addition is 0.0128 $ m^–2^ d^–1^, which is definitely counterbalanced by the value associated with the increase in nitrogen treatment efficiency. Therefore, alkalinity addition does make sense even from an economic point of view.

#### 3.7.3 Toward Advanced Process Control

The process optimization must be further explored considering a more complex problem with an efficiency criterion that combines algal production, nutrient removal rate, atmospheric emissions (NH_3_, CO_2_, N_2_O), and associated costs by simultaneously playing with alkalinity and the standard operating parameters (HRT, k_L_a, pH set point).

An advanced control problem similar to the one targeted in the work of de Luca et al.^37^ will help identify the optimal operational mode hourly adapted to the metereological conditions and online adjustment of the operational parameters. The ALBA model can now be used as a solid tool for process optimization while limiting emissions toward the environment.

## Supporting Information Available

SI.1: schematic representation of the raceway pond; SI.2: evaluation of experimental measurements’ standard deviations; SI.3: correlation between optical density at 680 nm and Total Suspended Solids concentration; SI.4: weather data set used in this study; SI.5: effect of temperature on the dissolved oxygen profile; SI.6: fractionation of the total biomass into algal and bacterial fractions; SI.7: calibration and validation of the temperature submodel; SI.8: description of the pH control implementation in the model; SI.9: computation of alkalinity and its effect on inorganic carbon availability; SI.10: extended scenario analysis results; SI.11: complete description of the ALBA model; SI.12: experimental measurement of k_L_a; SI.13 parameter uncertainty and error propagation; SI.14 residual analysis; SI.15 economic computation for alkalinity addition; SI.16 references (PDF)
