# Supplementary material for: Balancing Microalgae and Nitrifiers for Wastewater Treatment: Can Inorganic Carbon Limitation Cause an Environmental Threat?
Source: Environ Sci Technol. 2021 Mar 3;55(6):3940–55. doi: 10.1021/acs.est.0c05264 (PMC8028045; doi:10.1021/acs.est.0c05264)
Supplement: Supplementary file 1 — es0c05264_si_001.pdf [file es0c05264_si_001.pdf]

Balancing microalgae and nitrifiers for wastewater treatment: can inorganic carbon limitation cause an environmental threat?

Supporting Information

Francesca Casagli[a], Simone Rossi[a], Jean Philippe Steyer[b], Olivier Bernard[c], Elena Ficara[a,*]

[a]: Dipartimento di ingegneria civile e ambientale (DICA), Politecnico di Milano, 32, Piazza L. da Vinci, 20133 Milan, Italy.

[b]: Institut national de recherche pour l'agriculture, l'alimentation et l'environnement (INRAE), Univ Montpellier, LBE, 102, Avenue des étangs, 11100 Narbonne, France.

[c]: Institut national de recherche en informatique et en automatique (INRIA), Biocore, Université Cote d'Azur, 2004, Route des Lucioles – BP 93, 06902 Sophia Antipolis Cedex Sophia-Antipolis, France

[*]: Corresponding author: elena.ficara@polimi.it

9 Figures
14 Tables
43 Pages

SI.1 Pilot-scale raceway layout

A)

B)

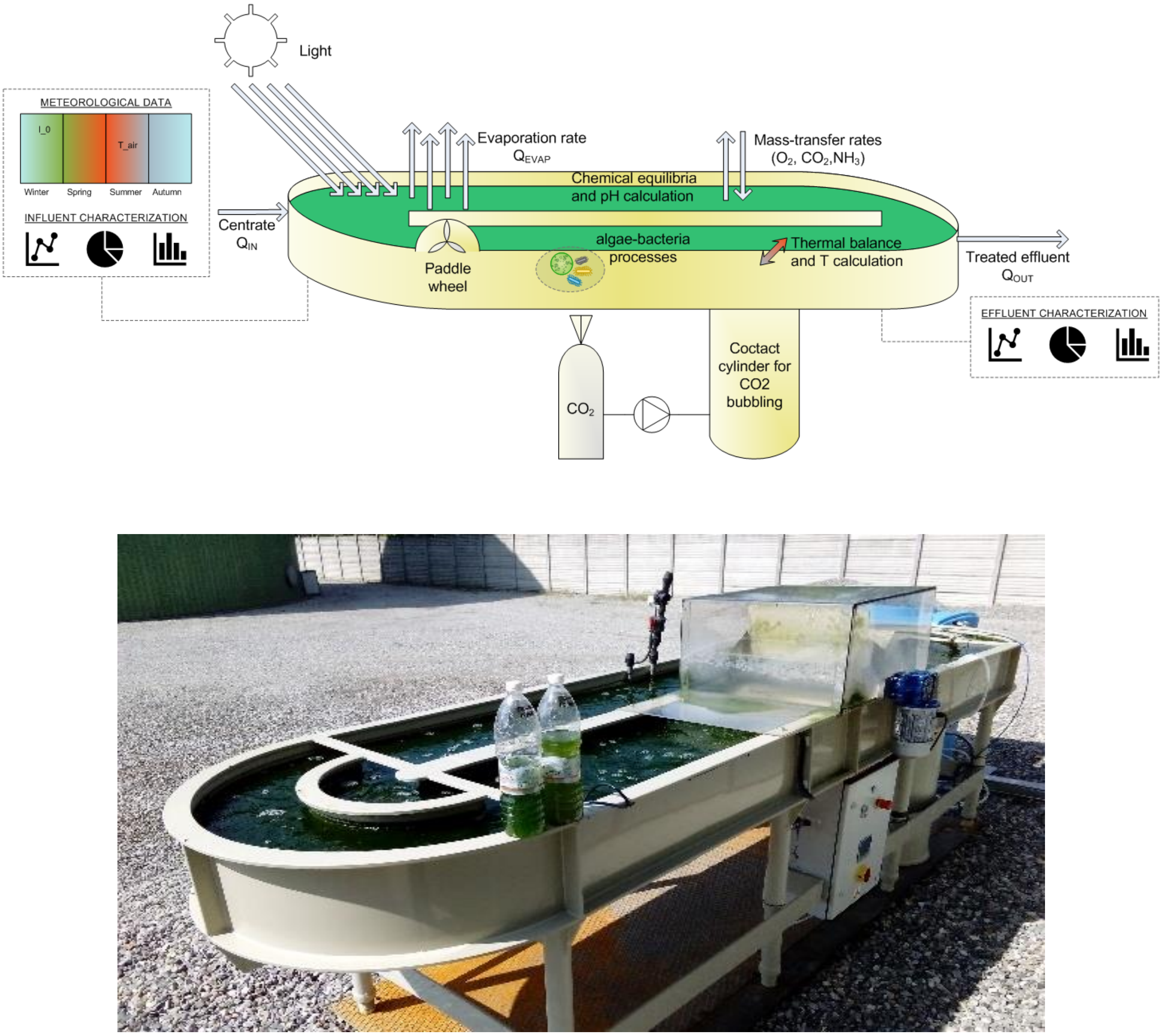

Figure SI.1.1. Scheme of the pilot-scale HRABP setup (A); picture of the pilot-scale HRABP (B).

SI.2 Evaluation of experimental measurements uncertainty

The standard deviation was estimated from the variation coefficient presented in Table SI.1.1. For measurements lower than a threshold ϖ the standard deviation is assumed to be constant.

Table SI.2.1. Measurement uncertainty modelling: standard deviation as a function of the mean value ω.

Measurement	Unit	Threshold	Standard deviation	
		ϖ	$\omega<\varpi$	$\varpi<\omega$
DO	$mgO_2.L^{-1}$	-	-	5% ϖ
pH	-	-	-	2% ϖ
sCOD	$mgCOD\ L^{-1}$	5	1	20% ϖ
COD_{ALG}	$mgCOD\ L^{-1}$	5	1	20% ϖ
$P\text{-}PO_4^{3-}$	$mgP\ L^{-1}$	5	1	20% ϖ
TAN	$mgN\ L^{-1}$	5	1	20% ϖ
$N\text{-}NO_3^-$	$mgN\ L^{-1}$	5	1	20% ϖ
$N\text{-}NO_2$	$mgN\ L^{-1}$	5	1	20% ϖ

SI.3 Correlation between optical density (680 nm) and TSS concentration

To determine the experimental algal biomass concentration, expressed in COD (X_{ALG_meas} in Fig.2C), the correlation factor among the OD_{680} and the TSS concentration was evaluated (Fig. SI 2.1), first multiplying the value of each TSS measurement for the conversion factor of 1.57 gCOD gBM_{ALG}^{-1} (from the stoichiometry) and for the ratio 0.87 gVSS $gTSS^{-1}$ (from experimental measurements). Thus, it was assumed that all the VSS were made of algal biomass. This hypothesis was done since experimental data of biomass fractionation in the raceway were not available. The model showed that the simulated algal biomass fraction was 90.2% of the total active biomass, on average (including bacteria, see Fig.SI.6.1 A).

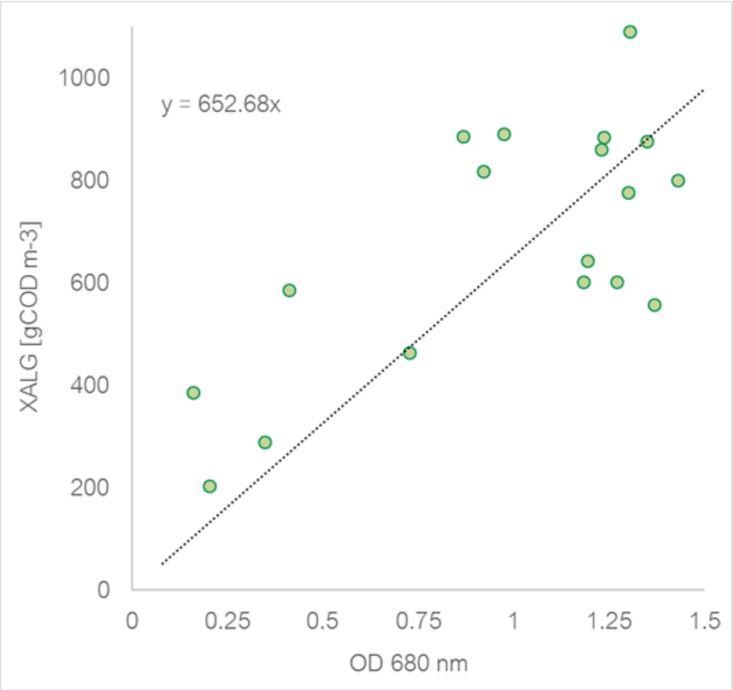

Figure SI.3.1. Correlation between the optical density at 680 nm (OD_{680}) and the algal biomass concentration (X_{ALG}, g COD m^{-3}) for the Milan case study.

SI.4 Weather dataset

A) B) C) D) E)

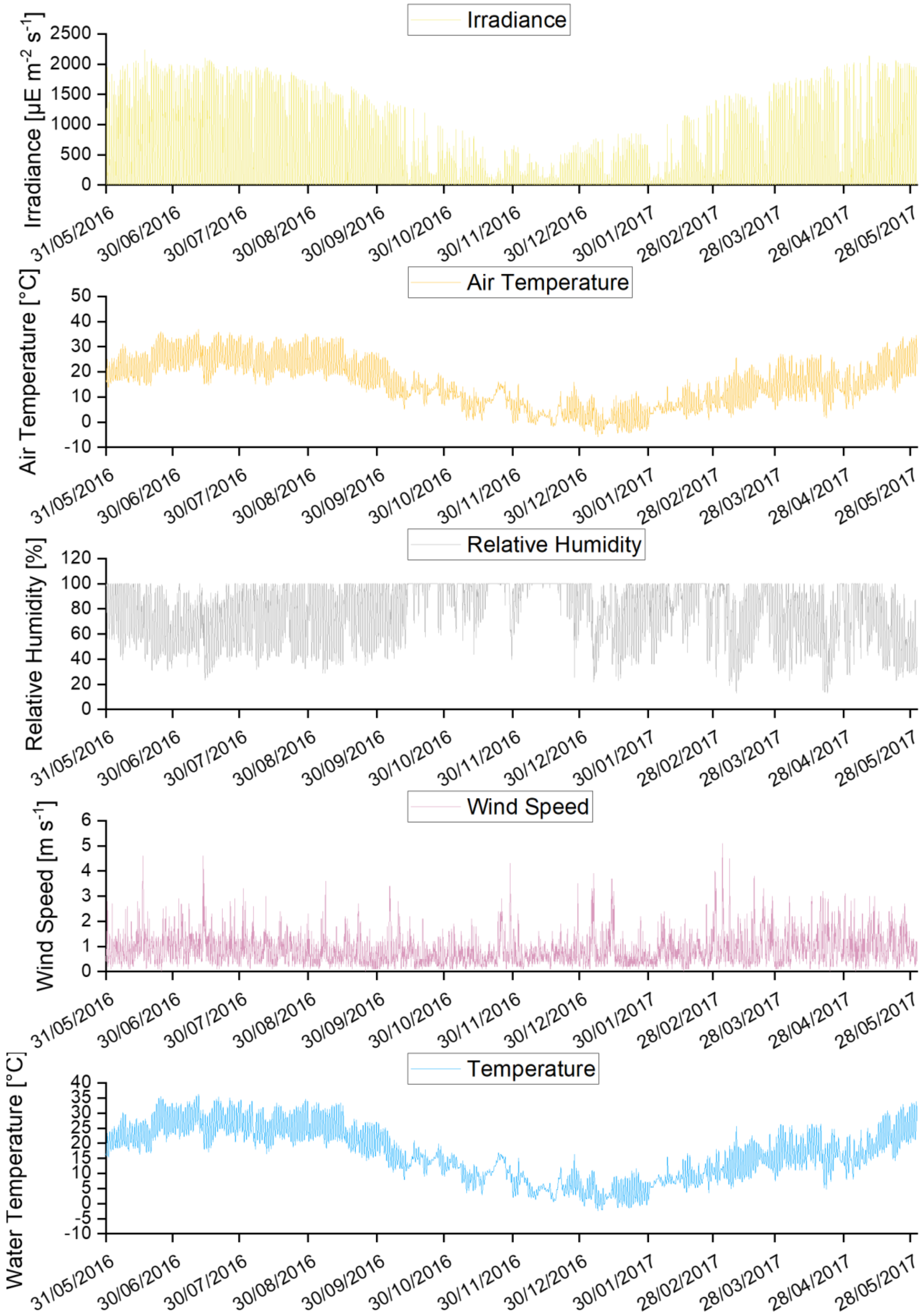

F) G)

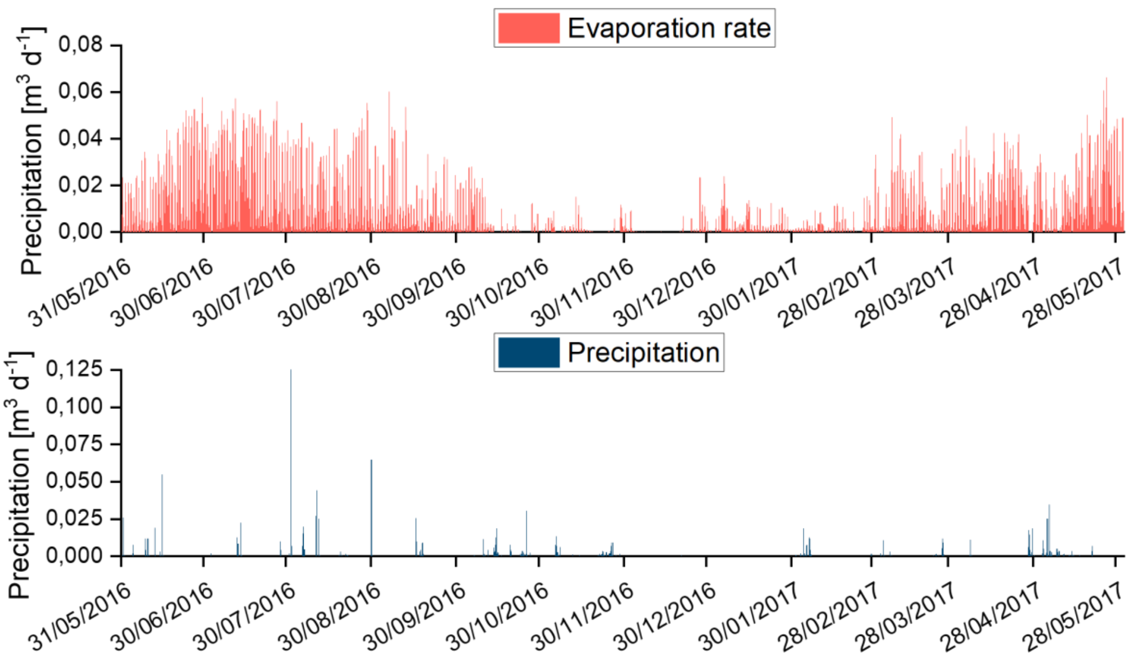

Figure SI.4.1. Weather dataset used for simulations: irradiance (A), air temperature (B), relative humidity (C), wind speed (D), pond temperature (E), evaporation rate (F), precipitation (G).

SI.5 Temperature effect on oxygen profile

Simulations were run to better understand the role played by temperature variations on the daily DO profile (Fig. SI.4.1). In addition to the results obtained running the model with the simulated temperature, simulations were run at the constant temperatures of 26 °C and 6°C (average temperature measured in summer and winter respectively). It can be clearly seen that the DO predictions obtained without accounting for temperature variations are not able to catch these dynamics. Indeed, only in the case where temperature affects DO solubility, the positive slope of the DO curve observed during the night can be effectively reproduced, while in all other cases, the DO curve is flat, or characterized by a negative slope.

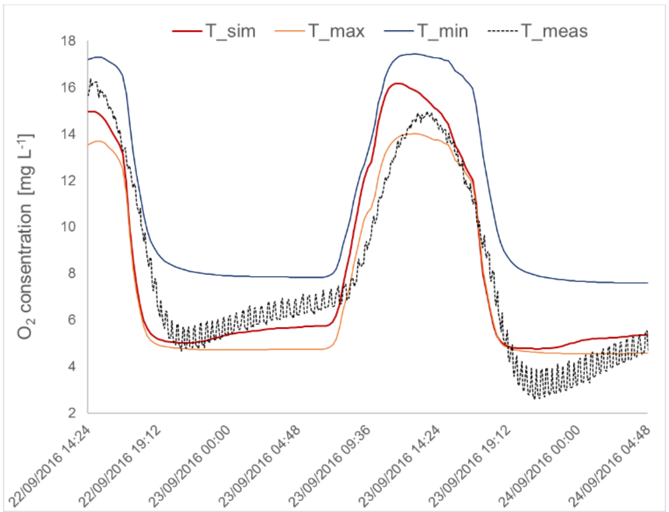

Figure SI.5.1. Temperature effect on dissolved oxygen daily dynamics: comparison between measured DO (black dotted line), simulated DO profiles under real dynamic temperature conditions (red line), constant temperature at 26°C (orange line), constant temperature at 6°C (blue line).

SI.6 Biomass fractionation

The biomass fractionation in the system, expressed in percentage, has been computed. In the present case study (Milan, I), where the 1 m^3 raceway was fed with diluted swine centrate, the system was mainly autotrophic, due to the influent characteristics (high N,P concentration and COD mainly recalcitrant) and the pH-control with CO_2 injection.

On the contrary, in the experimentation carried out in Casagli et al.[1] (Narbonne, FR), with 17 m^3 raceway fed with synthetic medium (simulating a municipal wastewater) the resulting biomass fractionations highlighted that heterotrophic bacteria were colonizing the system, together with algae, while nitrifiers constituted just the 1.44 % of the total biomass in average. Influent characteristics (high biodegradable organic matter) and no pH control active (source of additional inorganic carbon in the system), are the main responsible of the very low fraction of nitrifiers developed.

A)

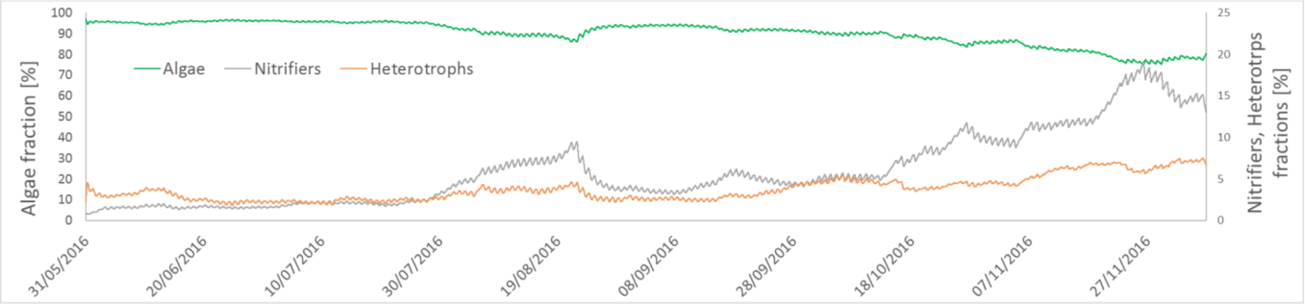

B)

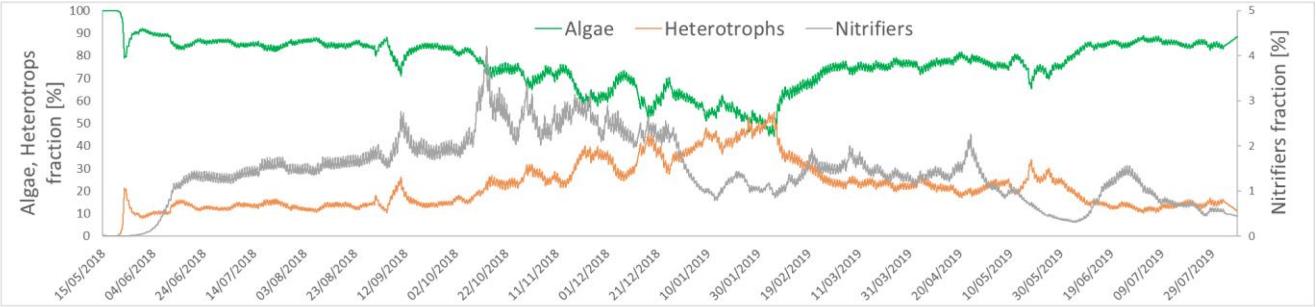

Figure SI.6.1. Biomass fractionation in percentage simulated by the model, divided among algae, heterotrophic bacteria and nitrifying bacteria (AOB+NOB). A): Milan case study, swine diluted centrate as influent. B): Narbonne case study, synthetic wastewater (reproducing a municipal one).

SI.7 Temperature sub-model: calibration and validation

A simple autoregressive model was developed to predict the temperature in the water ($T_{POND}(t)$) at time *t* from the measurement of the temperature in the air $\cdot T_{AIR}(t)$ at the same time *t* and from the temperature in the air at time t-Δ, $\cdot T_{AIR}(t-\Delta)$, where Δ is a time delay. A linear regression was computed for a range of delays Δ, and the delay providing the best regression (for Δ=4 hours) was finally selected.

The best regression was associated to R^2=0.98, given by:

$$T_{POND}(t)=0.46 \cdot T_{AIR}(t)+0.49 \cdot T_{AIR}(t-4)+2.18$$

$T_{AIR}(t-4)$ is the air temperature measured 4 hours before the time *t*.

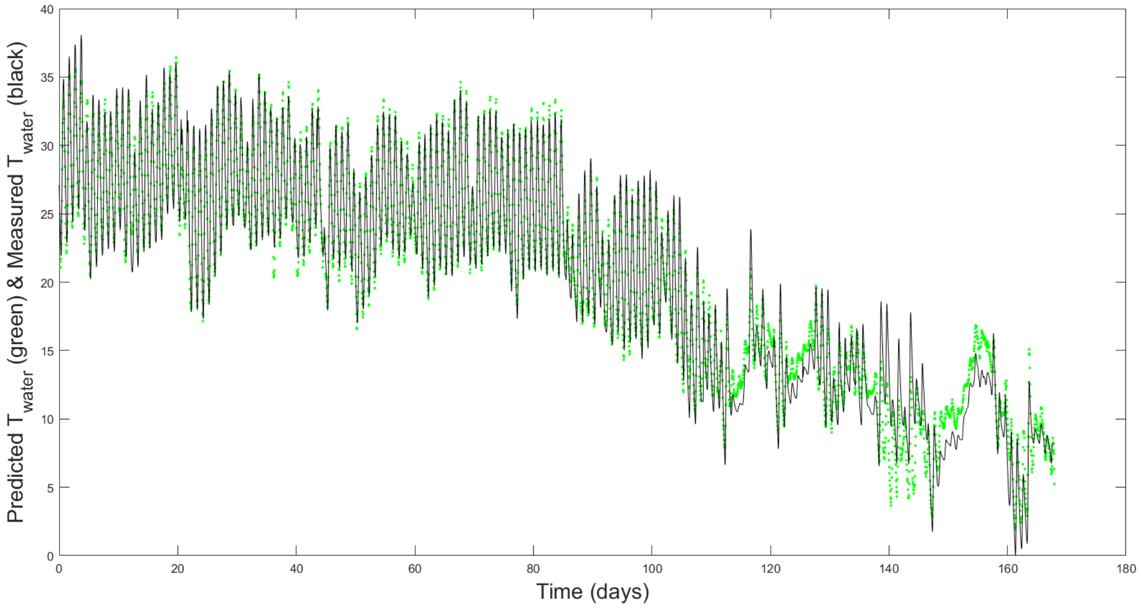

Figure SI.7.1. Measured and predicted pond temperature (R^2=0.98); the black line represents the measured pond temperature; the light green line represents the simulated pond temperature.

SI.8 pH control implementation

The pH control system was implemented in the ALBA model[1] as reported below (Eq. SI.8.1):

$$Rate_{pH_{control}}[gC\ m^{-3}d^{-1}]=\begin{cases} \Delta_{pH}\cdot\theta^{T-20}\cdot kLa\cdot\left(\frac{D_{CO2}}{D_{O2}}\right)^{0.5}\cdot\left(H_{CO2}(T)\cdot p_{CO2}-CO_2\right) & \Delta_{pH}>0 \\ 0 & \Delta_{pH}\leq 0 \end{cases}$$

where:

Δ_{pH}= (pH_s-pH_{tMAX}) is the difference between the simulated pH and the value set as maximun threshold reachable in the system for which the injection of CO_2 does not start.

D_{CO2} and D_{O2} are the diffusion coefficients of CO_2 and O_2 respectively [$m^2\ s^{-1}$]; k_La is the mass transfer coefficient [d^{-1}]; $H_{CO2}(T)$ is the Henry constant for CO_2 with temperature correction [gC-CO_2 m^{-3} d^{-1} atm^{-1}]; p_{CO2} is the partial pressure of CO_2 (set as 1 atm, since during the experimentation pure CO_2 was injected for controlling pH); CO_2 represents the concentration in the bulk volume [gC-CO_2 m^{-3}]; θ is the temperature correction factor for the mass transfer coefficient k_La.

In practice, the pH-control is modelled in a similar way as the CO_2 gas-liquid exchange with atmosphere but considering a different pressure of the gas and proportionally to the difference between pH in the raceway and the maximum pH allowed in the pond.

SI.9 Alkalinity

Total Alkalinity (TA) was computed according to Dickson[2] and Wolf-Gladrow et al.[3] as reported below (Eq. SI.9.1):

$$TA = HCO_3^- + 2CO_3^{2-} + H_2PO_4^- + HPO_4^{2-} + 2PO_4^{3-} + OH^- + NH_3 - H^+ - HNO_2 - HNO_3 - H_3PO_4 \qquad \text{SI.9.1}$$

The digestate can present significant concentrations of volatile fatty acids (VFA - acetate, butyrate, propionate and valerate) and hydrogen sulphide. In this case, the Total Alkalinity formula should account also for them, as reported below (Eq. SI.9.2):

$$TA = HCO_3^- + 2CO_3^{2-} + H_2PO_4^- + HPO_4^{2-} + 2PO_4^{3-} + OH^- + NH_3 + C_2H_3OO^- + C_4H_7OO^- + C_3H_5OO^- + C_5H_9OO^- + HS^- + 2S^{2-} - H^+ - HNO_2 - HNO_3 - H_3PO_4 \qquad \text{SI.9.2}$$

Dynamic simulations (see Fig.2) were first run at pH controlled with CO_2 injection at a setpoint of 7.5, well known for guarantying not limiting CO_2 conditions for algae and nitrifiers growth. However, the model revealed dissolved inorganic carbon limitation conditions, as shown in Fig. SI.9.1 (line S_IC). Indeed, a close look at alkalinity (Fig. SI.9.1, line TA) highlights a regular drop, due to both the exhaustion of ammonium and the production of nitrate and nitrite (see Fig. 2A and 2B) . The resulting very low alkalinity does not allow to store bicarbonate in solution. More generally, the level of dissolved inorganic carbon remains definitely limiting and therefore a strong competition for inorganic carbon between algae and nitrifiers occurs.

Additional simulations were run increasing the influent alkalinity concentration of 10 mol m^{-3} (TA_2), 15 mol m^{-3} (TA_2.5) and 20 mol m^{-3} (TA_3), tuning the variable Δ_{CAT,AN_IN} (by multiplying it by a factor of 2, 2.5 and 3) to reproduce this effect and maintaining a regulated

pH at 7.5. Results are shown in Fig. SI.9.1, in terms of TA concentration (mol m^{-3}) and the corresponding S_IC concentration (mgC L^{-1}). It is evident that increasing the influent alkalinity allows to store considerably higher concentration of dissolved inorganic carbon, solving issues as the competition between algae and nitrifiers and the favourable conditions for N_2O production and emission.

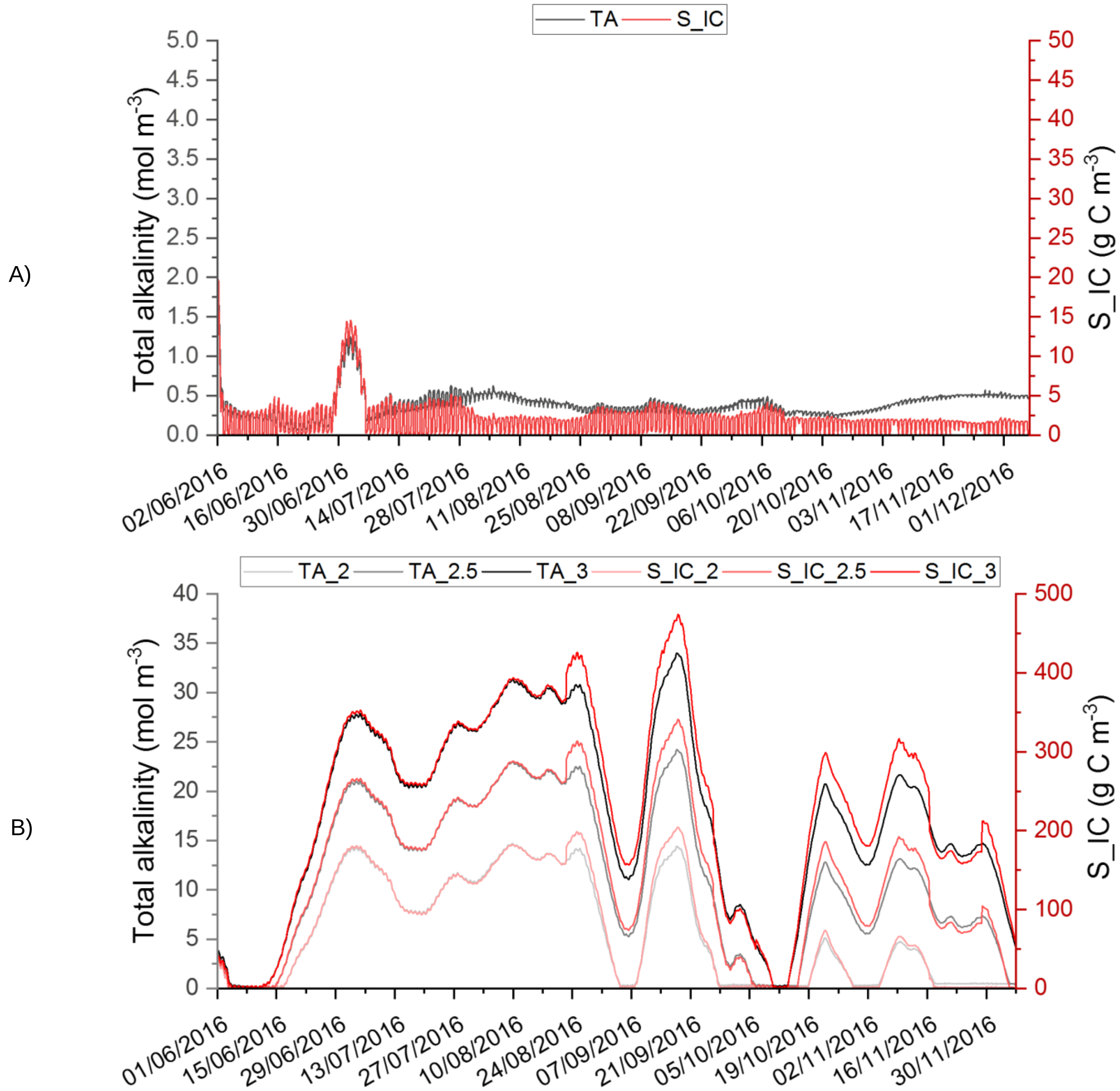

Figure SI.9.1. Dynamic simulations of alkalinity (left axis) and inorganic carbon (S_IC, right axis) at different influent alkalinity concentrations: A) Reference case (S1); B) simulations at influent alkalinity increased by 10, 15 and 20 mol m^{-3}, respectively. The alkalinity is computed as in Eq. SI.9.1.

SI.10 Scenarios analysis results

Table SI.10.1. Simulation results describing the system performances in spring.

RESULTS: SPRING												
Parameter tested	HRT	pH	k_La	X_ALG_prod	APPARENT SNH_REM_RATE	ACTUAL SNH_REM_RATE	SPO_4 REM_RATE	CO_2 TRAN_RATE(**)	S_IC	NH_3 TRAN_RATE	N_2O_f	Scenario
-	[d]	[-]	[d^{-1}]	[gTSS $m^{-2} d^{-1}$]	[gN-TAN $m^{-2}d^{-1}$]	[gN-TAN $m^{-2}d^{-1}$]	[gP-PO_4^{3-} $m^{-2}d^{-1}$]	[gC-CO_2 $m^{-3}d^{-1}$]	[gC m^{-3}]	[gN-NH_3 m^{-2} d^{-1}]	[%]	n°
k_La, HRT	10	7.5	34	12.28	6.91	5.74	0.18	-1.63	1.67	-1.18	56	1
	10	7.5	0.5	13.38	7.04	7.04	0.20	1.63	8.69	-0.01	27	2
HRT	2	7.5	34	22.09	20.08	1.75	0.29	24.55	256.48	-18.33	0	3
	2	7	34	21.60	10.31	1.69	0.28	74.52	360.02	-8.62	0	3.1
	2	6.5	34	21.13	4.83	1.64	0.28	122.84	459.43	-3.18	0	3.2
	5	7.5	34	18.34	13.13	11.41	0.27	-17.97	3.4	-1.72	45	4
	5	7.5	0.5	19.49	11.24	11.19	0.27	-2.43	76.8	-0.05	0	4.1
	15	7.5	34	8.87	4.73	3.86	0.13	0.83	1.39	-0.86	60	5
	20	7.5	34	6.56	3.60	2.90	0.10	0.79	1.28	-0.70	61	6
pH	5	6.5	34	17.37	12.28	12.28	0.26	-20.31	2.94	0.00	46	7
	5	7	34	18.02	12.29	12.29	0.27	-19.09	2.91	0.00	47	8
	5	8	34	18.03	14.42	9.94	0.26	-19.57	4.22	-4.49	41	9
	5	NC	34	15.47	15.10	8.94	0.22	-24.79	4.75	-6.16	39	10
TA(*)	5	7.5	34	18.83	16.29	16.29	0.29	8.37	132.13	0.00	0	11
pH	10	6.5	34	12.29	6.33	6.33	0.18	-2.22	1.44	0.00	63	12
	10	7	34	12.51	6.34	6.34	0.18	-1.54	1.50	0.00	60	13
	10	8	34	11.42	7.58	4.97	0.17	-4.38	1.99	-2.61	53	14
	10	NC	34	6.92	7.91	4.18	0.10	-14.05	2.06	-3.73	54	15

(*)TA=Total Alkalinity. In this scenario, the concentration of TA (expressed in mol m^{-3}) in the influent was increased by 20 mol m^{-3}.
(**)The CO_2 transfer rate is expressed for unit of volume. To obtain the surface transfer rate, it' sufficient to divide the value for the raceway surface (3.8 m^2), since the reactor volume was 1 m^3.

Table SI.10.2. Simulation results describing the system performances in summer.

RESULTS: SUMMER												
Effect tested	HRT	pH	k_La	X_ALG prod	APPARENT SNH_REM_RATE	ACTUAL SNH_REM_RATE	SPO_4 REM_RATE	CO_2 TRAN_RATE(**)	S_IC	NH_3 TRAN_RATE	N_2O_f	Scenario
-	[d]	[-]	[d^{-1}]	[gTSS m^{-2} d^{-1}]	[gN-TAN $m^{-2}d^{-1}$]	[gN-TAN $m^{-2}d^{-1}$]	[gP-PO_4^{3-} $m^{-2}d^{-1}$]	[gC-CO_2 $m^{-3}d^{-1}$]	[gC m^{-3}]	[gN-NH_3 m^{-2} d^{-1}]	[%]	n°
k_La, HRT (C, N, O partitioning)	10	7.5	34	11.70	7.22	5.44	0.17	-2.3	2.1	-1.78	54	1
	10	7.5	0.5	13.11	7.04	7.01	0.20	1.8	9.3	-0.03	23	2
HRT	2	7.5	34	21.05	24.26	1.67	0.27	7.69	227.47	-22.59	0	3
	2	7	34	20.43	14.33	1.60	0.26	56.01	328.68	-12.72	0	3.1
	2	6.5	34	19.85	8.00	1.55	0.26	104.27	428.71	-6.45	0	3.2
	5	7.5	34	17.26	13.61	10.76	0.25	-20.05	4.1	-2.85	42	4
	5	7.5	0.5	11.55	6.67	6.51	0.15	8.27	224	-0.16	0	4.1
	15	7.5	34	8.38	4.93	3.69	0.13	0.63	1.70	-1.24	56	5
	20	7.5	34	6.15	3.76	2.80	0.10	0.90	1.57	-0.95	58	6
pH	5	6.5	34	16.24	12.24	12.24	0.24	-22.09	4.05	0.00	32	7
	5	7	34	16.90	12.42	12.08	0.25	-20.94	3.37	-0.34	46	8
	5	7	34	17.40	37.96	27.36	0.24	-110.01	144.81	-10.61	0	8.1
	5	8	34	17.13	14.84	9.35	0.24	-20.74	5.44	-5.49	36	9
	5	NC	34	15.98	15.15	8.90	0.22	-23.17	5.80	-6.25	35	10
TA	5	7.5	34	17.54	16.25	16.25	0.27	5.89	133.16	0.00	0	11
pH	10	6.5	34	11.45	6.33	6.33	0.17	-3.33	1.47	0.00	65	12
	10	7	34	11.70	6.46	6.21	0.17	-2.63	1.61	-0.25	58	13
	10	8	34	11.42	7.75	4.85	0.17	-3.25	2.61	-2.90	50	14
	10	NC	34	7.06	8.08	4.04	0.10	-13.26	2.88	-4.05	51	15

(*)TA=Total Alkalinity. In this scenario, the concentration of TA (expressed in mol m^{-3}) in the influent was increased by 20 mol m^{-3}.
(**)The CO_2 transfer rate is expressed for unit of volume. To obtain the surface transfer rate, it' sufficient to divide the value for the raceway surface (3.8 m^2), since the reactor volume was 1 m^3.

Table SI.10.3. Simulation results describing the system performances in autumn.

RESULTS: AUTUMN												
Effect tested	HRT	pH	k_La	X_ALG prod	APPARENT SNH_REM_RATE	ACTUAL SNH_REM_RATE	SPO_4 REM_EFF	CO_2 TRAN_RATE(**)	S_IC	NH_3 TRAN_RATE	N_2O_f	Scenario
-	[d]	[-]	[d^{-1}]	[gTSS m^{-2} d^{-1}]	[gN-TAN $m^{-2}d^{-1}$]	[gN-TAN $m^{-2}d^{-1}$]	[gP-PO_4^{3-} $m^{-2}d^{-1}$]	[gC-CO_2 m^{-3} d^{-1}]	[gC m^{-3}]	[gN-NH_3 m^{-2} d^{-1}]	[%]	n°
k_La, HRT (C, N, O partitioning)	10	7.5	34	5.96	6.00	6.00	0.09	-15.7	1.8	0	49	1
	10	7.5	0.5	8.80	5.58	5.58	0.13	-1.70	87.10	0.00	0	2
HRT	2	7.5	34	7.27	8.49	0.80	0.11	37.47	335.08	-7.69	0	3
	2	8	34	7.62	19.98	0.85	0.12	-9.41	239.61	-19.14	0	3.1
	2	6.5	34	6.12	0.71	0.71	0.10	122.99	510.59	0.00	0	3.2
	5	7.5	34	8.63	11.84	11.84	0.145	-37.4	4.5	0	22	4
	5	7.5	0.5	6.21	3.09	2.94	0.08	17	315.6	-0.15	0	4.1
	15	7.5	34	4.22	4.02	4.02	0.06	-10.23	1.30	0.00	100	5
	20	7.5	34	2.97	3.01	3.01	0.04	-7.85	1.06	0.00	100	6
pH	5	6.5	34	8.22	11.79	11.79	0.140	-37.46	8.19	0.00	0	7
	5	7	34	8.57	11.85	11.85	0.144	-37.68	4.45	0.00	23	8
	5	8	34	8.63	11.93	11.75	0.144	-37.51	4.65	-0.18	21	9
	5	NC	34	8.63	11.93	11.75	0.144	-37.51	4.65	-0.18	21	10
TA	5	7.5	34	9.01	16.29	16.29	0.16	-14.45	110.75	0.00	0	11
pH	10	6.5	34	6.32	6.03	6.03	0.10	-15.06	1.79	0.00	53	12
	10	7	34	6.33	6.02	6.02	0.10	-15.08	1.79	0.00	51	13
	10	8	34	4.98	6.45	5.46	0.08	-18.16	1.85	-0.99	42	14
	10	NC	34	4.49	6.51	5.36	0.07	-19.10	1.84	-1.15	43	15

(*)TA=Total Alkalinity. In this scenario, the concentration of TA (expressed in mol m^{-3}) in the influent was increased by 20 mol m^{-3}.
(**)The CO_2 transfer rate is expressed for unit of volume. To obtain the surface transfer rate, it' sufficient to divide the value for the raceway surface (3.8 m^2), since the reactor volume was 1 m^3.

Table SI.10.4. Simulation results describing the system performances in winter.

RESULTS: WINTER													
Effect tested	HRT	pH	k_La	X_ALG prod	APPARENT SNH_REM_RATE	ACTUAL SNH_REM_RATE	SNH REM_EFF	SPO4 REM_EFF	CO_2 TRAN_RATE(**)	S_IC	NH_3 TRAN_RATE	N_2O_f	Scenario
-	[d]	[-]	[d^{-1}]	[gTSS m^{-2} d^{-1}]	[gN-TAN $m^{-2}d^{-1}$]	[gN-TAN $m^{-2}d^{-1}$]	[%]	[gP-PO_4^{3-} $m^{-2}d^{-1}$]	[gC-CO_2 $m^{-3}d^{-1}$]	[gC m^{-3}]	[gN-NH_3 m^{-2} d^{-1}]	[%]	n°
k_La, HRT (C, N, O partitioning)	10	7.5	34	6.44	6.01	6.01	73.56	0.10	-14.3	2.6	0	35	1
	10	7.5	0.5	7.15	3.87	3.87	47.37	0.10	6.10	202.10	0.00	0	2
HRT	2	7.5	34	0.00	3.31	0.39	0.96	0.03	43.97	373.69	-2.92	0	3
	2	6.5	34	0.00	0.38	0.38	0.93	0.03	123.24	532.14	0.00	0	3.1
	5	7.5	34	8.41	10.09	10.09	61.75	0.145	-26.1	62.1	0.00	0	4
	5	7.5	0.5	4.48	1.39	1.32	8.08	0.06	22.2	356.7	-0.07	0	4.1
	15	7.5	34	4.74	4.04	4.04	74.11	0.07	-8.91	1.66	0.00	64	5
	20	7.5	34	3.60	3.03	3.03	74.30	0.06	-6.32	1.31	0.00	100	6
pH	5	6.5	34	6.72	0.56	0.56	3.42	0.09	60.85	527.22	0.00	0	7
	5	7	34	6.83	0.58	0.58	3.53	0.09	43.18	439.77	0.00	0	8
	5	8	34	8.61	11.44	10.89	66.66	0.151	-33.11	24.10	-0.55	0	9
	5	NC	34	8.61	11.47	10.87	66.54	0.151	-33.15	23.84	-0.60	0	10
TA	5	7.5	34	8.43	10.52	10.52	64.37	0.15	22.93	307.87	0.00		11
pH	10	6.5	34	6.30	6.01	6.01	73.57	0.10	-14.65	3.23	0.00	27	12
	10	7	34	6.49	6.02	6.02	73.65	0.10	-14.33	2.53	0.00	38	13
	10	8	34	6.04	6.19	5.78	70.70	0.10	-15.19	2.71	-0.42	33	14
	10	NC	34	5.42	6.46	5.44	66.64	0.09	-16.69	2.90	-1.01	32	15

(*)TA=Total Alkalinity. In this scenario, the concentration of TA (expressed in mol m^{-3}) in the influent was increased by 20 mol m^{-3}.

(**)The CO_2 transfer rate is expressed for unit of volume. To obtain the surface transfer rate, it' sufficient to divide the value for the raceway surface (3.8 m^2), since the reactor volume was 1 m^3.

SI.11 The ALBA model

Table SI.11.1. Stoichiometric matrix of the ALBA model (Casagli et al.[1]).

component j → process i ↓		X_{ALG} gCOD m^{-3}	X_{AOB} gCOD m^{-3}	X_{NOB} gCOD m^{-3}	X_H gCOD m^{-3}	X_S gCOD m^{-3}	X_I gCOD m^{-3}	S_S gCOD m^{-3}	S_I gCOD m^{-3}	S_{IC} gCm^{-3}	S_{ND} gNm^{-3}	S_{NH} gNm^{-3}	S_{NO2} gNm^{-3}	S_{NO3} gNm^{-3}	S_{N2} gNm^{-3}	S_{PO4} gPm^{-3}	S_{O2} gO$_2$m^{-3}	S_{H2O} gHm^{-3}
Algae																		
1	phototrophic growth on NH_4^+	1								$\alpha_{1,9}$		$\alpha_{1,11}$				$\alpha_{1,15}$	1	$\alpha_{1,17}$
2	phototrophic growth on NO_3^-	1								$\alpha_{2,9}$				$\alpha_{2,13}$		$\alpha_{2,15}$	1	$\alpha_{2,17}$
3	aerobic respiration	-1								$\alpha_{3,9}$		$\alpha_{3,11}$				$\alpha_{3,15}$	-1	$\alpha_{3,17}$
4	Decay	-1				$\alpha_{4,5}$	$\alpha_{4,6}$			$\alpha_{4,9}$		$\alpha_{4,11}$				$\alpha_{4,15}$		
Heterotrophic bacteria																		
5	Aerobic growth on NH_4^+				1			$\alpha_{5,7}$		$\alpha_{5,9}$		$\alpha_{5,11}$				$\alpha_{5,15}$	$\alpha_{5,16}$	
6	Aerobic growth on NO_3^-				1			$\alpha_{6,7}$		$\alpha_{6,9}$				$\alpha_{6,13}$		$\alpha_{6,15}$	$\alpha_{6,16}$	
7	Aerobic respiration				-1					$\alpha_{7,9}$		$\alpha_{7,11}$				$\alpha_{7,15}$	-1	
8	Anoxic growth on NO_3^-				1			$\alpha_{8,7}$		$\alpha_{8,9}$		$\alpha_{8,11}$		$\alpha_{8,13}$	$\alpha_{8,14}$	$\alpha_{8,15}$		
9	Anoxic growth on NO_2^-				1			$\alpha_{9,7}$		$\alpha_{9,9}$		$\alpha_{9,11}$	$\alpha_{9,12}$		$\alpha_{9,14}$	$\alpha_{9,15}$		
10	Anoxic respiration NO_2^- and NO_3^-				-1					$\alpha_{10,9}$		$\alpha_{10,11}$	$\alpha_{10,12}$	$\alpha_{10,13}$	$\alpha_{10,14}$	$\alpha_{10,15}$		
11	Hydrolysis of slowly biodegradable COD					-1		$\alpha_{11,7}$	$\alpha_{11,8}$	$\alpha_{11,9}$		$\alpha_{11,11}$				$\alpha_{11,15}$		
12	Hydrolysis of urea									$\alpha_{12,9}$	-1	1						$\alpha_{12,17}$
13	Decay				-1	$\alpha_{13,5}$	$\alpha_{13,6}$			$\alpha_{13,9}$		$\alpha_{13,11}$				$\alpha_{13,15}$		
Ammonium Oxydising Bacteria																		
14	Aerobic growth on NH_4^+		1							$\alpha_{14,9}$		$\alpha_{14,11}$	$\alpha_{14,12}$			$\alpha_{14,15}$	$\alpha_{14,16}$	
15	Aerobic respiration		-1							$\alpha_{15,9}$		$\alpha_{15,11}$				$\alpha_{15,15}$	$\alpha_{15,16}$	

16	Decay		-1			$\alpha_{16,5}$	$\alpha_{16,6}$			$\alpha_{16,9}$		$\alpha_{16,11}$				$\alpha_{16,15}$		
Nitrite Oxydising Bacteria																		
17	Aerobic growth on NO_2^-			1						$\alpha_{17,9}$		$\alpha_{17,11}$	$\alpha_{17,12}$	$\alpha_{17,13}$		$\alpha_{17,15}$	$\alpha_{17,16}$	
18	Aerobic respiration			-1						$\alpha_{18,9}$		$\alpha_{18,11}$				$\alpha_{18,15}$	$\alpha_{18,16}$	
19	Decay			-1		$\alpha_{19,5}$	$\alpha_{19,6}$			$\alpha_{19,9}$		$\alpha_{19,11}$				$\alpha_{19,15}$		
Equilibrium phase																		
20	Dissolution of O_2																1	
21	Dissolution of CO_2									1								
22	Dissolution of NH_3											1						
k ↓	j→								Composition matrix									
1	COD (4 → 19)	1	1	1	1	1	1	1	1	0	0	0	-3.43	-4.57	-1.71	0	-1	0
2	O (1 → 3)	$iO_{BM,ALG}$	iO_{BM}	iO_{BM}	iO_{BM}	iO_{XS}	iO_{XI}	iO_{SS}	iO_{SI}	2.67	0.57	0	2.28	3.43	0	2.07	1	7.94
3	C	$iC_{BM,ALG}$	iC_{BM}	iC_{BM}	iC_{BM}	IC_{Xs}	iC_{xi}	iC_{ss}	iC_{si}	1	0.43	0	0	0	0	0	0	0
4	N	$iN_{BM,ALG}$	iN_{BM}	iN_{BM}	iN_{BM}	IN_{Xs}	iN_{xi}	iN_{ss}	iN_{si}	0	1	1	1	1	1	0	0	0
5	P	$iP_{BM,ALG}$	iP_{BM}	iP_{BM}	iP_{BM}	IP_{Xs}	iP_{xi}	iP_{ss}	iP_{si}	0	0	0	0	0	0	1	0	0
6	H (1 → 3)	$iH_{BM,ALG}$	iH_{BM}	iH_{BM}	iH_{BM}	0	0	0	0	0	0.14	0.22	0.07	0.07	0	0.10	0	1

Table SI.11.2. Stoichiometric coefficient values in the ALBA model (Casagli et al.[1]).

Symbol	Definition	Value	Unit	Source
		ALGAE - BACTERIA		
$f_{XI,ALG}$	Inert organic fraction produced from microalgae decay	0.062	$gCOD_{XI}\ gCOD_{BM}^{-1}$	1
$i_{C,BM}^{ALG}$	Fraction of carbon in algae biomass	0.327	$gC\ gCOD_{BM}^{-1}$	1
$i_{N,BM}^{ALG}$	Fraction of nitrogen in algae biomass	0.042	$gN\ gCOD_{BM}^{-1}$	1
$i_{P,BM}^{ALG}$	Fraction of phosphorus in algae biomass	0.008	$gP\ gCOD_{BM}^{-1}$	1
$i_{O,BM}^{ALG}$	Fraction of oxygen in algae biomass	0.209	$gO\ gCOD_{BM}^{-1}$	1
$i_{H,BM}^{ALG}$	Fraction of hydrogen in algae biomass	0.050	$gH\ gCOD_{BM}^{-1}$	1
f_{SI}	Inert soluble organic fraction produced from hydrolysis	0.1	$gCOD_{SI}\ gCOD_{BM}^{-1}$	4
f_{XI}	Inert organic fraction produced from bacteria decay	0.1	$gCOD_{XI}\ gCOD_{BM}^{-1}$	4
$i_{C,BM}$	Fraction of carbon in bacterial biomass (nitrifiers, heterotrophs)	0.36	$gC\ gCOD_{BM}^{-1}$	1
$i_{N,BM}$	Fraction of nitrogen in bacterial biomass (nitrifiers, heterotrophs)	0.084	$gN\ gCOD_{BM}^{-1}$	1
$i_{P,BM}$	Fraction of phosphorus in bacterial biomass (nitrifiers, heterotrophs)	0.016	$gP\ gCOD_{BM}^{-1}$	1
$i_{O,BM}$	Fraction of oxygen in bacterial biomass (nitrifiers, heterotrophs)	0.184	$gO\ gCOD_{BM}^{-1}$	1
$i_{H,BM}$	Fraction of hydrogen in bacterial biomass (nitrifiers, heterotrophs)	0.043	$gH\ gCOD_{BM}^{-1}$	1
$i_{C,SS}$	Fraction of carbon in soluble organic matter (S_S)	0.318	$gC\ gCOD_{SS}^{-1}$	5
$i_{N,SS}$	Fraction of nitrogen in soluble organic matter (SS)	0.015	$gN\ gCOD_{SS}^{-1}$	1
$i_{P,SS}$	Fraction of phosphorus in soluble organic matter (SS)	0.005	$gP\ gCOD_{SS}^{-1}$	5
$i_{O,SS}$	Fraction of oxygen in soluble organic matter (SS)	0.156	$gO\ gCOD_{SS}^{-1}$	5
$i_{C,SI}$	Fraction of carbon in soluble recalcitrant organic matter (SI)	0.36	$gC\ gCOD_{SI}^{-1}$	5
$i_{N,SI}$	Fraction of nitrogen in soluble recalcitrant organic matter (SI)	0.06	$gN\ gCOD_{SI}^{-1}$	5
$i_{P,SI}$	Fraction of phosphorus in soluble recalcitrant organic matter (SI)	0.005	$gP\ gCOD_{SI}^{-1}$	5
$i_{O,SI}$	Fraction of oxygen in soluble recalcitrant organic matter (SI)	0.15	$gO\ gCOD_{SI}^{-1}$	5
Y_H	Growth yield for heterotrophic bacteria	0.63	$gCOD_{BM}\ gCOD_{SS}^{-1}$	4
$Y_{H,NO2}$	Growth yield for heterotrophic bacteria, denitrification on NO_2^-	0.3	$gCOD_{BM}\ gCOD_{SS}^{-1}$	5
$Y_{H,NO3}$	Growth yield for heterotrophic bacteria, denitrification on NO_3^-	0.5	$gCOD_{BM}\ gCOD_{SS}^{-1}$	5
Y_{AOB}	Growth yield factor for AOB	0.2	$gCOD_{BM}\ gN^{-1}$	6
Y_{NOB}	Growth yield for NOB	0.05	$gCOD_{BM}\ gN^{-1}$	6
$i_{C,ND}$	Fraction of inorganic carbon from urea hydrolysis (S_{ND})	0.429	$gC\ gN_{urea}^{-1}$	1
$i_{H,ND}$	Fraction of hydrogen from urea hydrolysis (S_{ND})	0.072	$gH\ gN_{urea}^{-1}$	1
$i_{C,XS}$	Fraction of carbon in particulate biodegradable organic matter	0.318	$gC\ gCOD_{XS}^{-1}$	5
$i_{C,XI}$	Fraction of carbon in particulate inert organic matter	0.36	$gC\ gCOD_{XI}^{-1}$	7

$i_{N,XS}$	Fraction nitrogen in particulate biodegradable organic matter	0.034	$gN\ gCOD_{XS}^{-1}$	5
$i_{N,XI}$	Fraction of nitrogen in particulate inert organic matter	0.06	$gN\ gCOD_{XI}^{-1}$	7
$i_{P,XS}$	Fraction of phosphorus in particulate biodegradable organic matter	0.005	$gP\ gCOD_{XS}^{-1}$	5
$i_{P,XI}$	Fraction of phosphorus in particulate inert organic matter	0.01	$gP\ gCOD_{XI}^{-1}$	4
$i_{O,XS}$	Fraction of oxygen in particulate biodegradable organic matter	0.156	$gO\ gCOD_{XS}^{-1}$	5
$i_{O,XI}$	Fraction of oxygen in particulate inert organic matter	0.15	$gO\ gCOD_{XI}^{-1}$	5

Table SI.11.3. Stoichiometric coefficient expressions in the ALBA model (Casagli et al.[1]).

Stoichiometric coefficients			
Symbol	Affected variable	Expression	Unit
ρ_1 - Growth of X_{ALG} on NH_4^+			
$\alpha_{1,1}$	X_{ALG}	1	$gCOD_{BM}$ / $gCOD_{BM}$
$\alpha_{1,9}$	S_{IC}	$- iC_{,BM}^{ALG}$	$gC/gCOD_{BM}$
$\alpha_{1,11}$	S_{NH}	$- iN_{,BM}^{ALG}$	$gN/gCOD_{BM}$
$\alpha_{1,15}$	S_{PO4}	$- iP_{,BM}^{ALG}$	$gP/gCOD_{BM}$
$\alpha_{1,16}$	S_{O2}	$- iO_{,BM}^{ALG}+(32/12)\ iC_{,BM}^{ALG}-(24/14)iN_{,BM}^{ALG}$ $+(40/31)\ iP_{,BM}^{ALG}+(8)\ iH_{,BM}^{ALG}$	$gO_2/gCOD_{BM}$
$\alpha_{1,17}$	S_{H2O}	- 0.0404	$gH/gCOD_{BM}$
ρ_2 - Growth of X_{ALG} on NO_3^-			
$\alpha_{2,1}$	X_{ALG}	1	$gCOD_{BM}$ / $gCOD_{BM}$
$\alpha_{2,9}$	S_{IC}	$- iC_{,BM}^{ALG}$	$gC/gCOD_{BM}$
$\alpha_{2,13}$	S_{NO3}	$- iN_{,BM}^{ALG}$	$gN/gCOD_{BM}$
$\alpha_{2,15}$	S_{PO4}	$- iP_{,BM}^{ALG}$	$gP/gCOD_{BM}$
$\alpha_{2,16}$	S_{O2}	$-iO_{BM}^{ALG}+(32/12)\ iC_{BM}^{ALG}+(40/14)\ iN_{BM}^{ALG}$ $+(40/31)\ iP_{BM}^{ALG}+(8)\ iH_{BM}^{ALG}$	$gO/gCOD_{BM}$
$\alpha_{2,17}$	S_{H2O}	- 0.0464	$gH/gCOD_{BM}$
ρ_3 – Aerobic respiration of X_{ALG}			
$\alpha_{3,1}$	X_{ALG}	-1	$gCOD_{BM}$ / $gCOD_{BM}$
$\alpha_{3,9}$	S_{IC}	$iC_{,BM}^{ALG}$	$gC/gCOD_{BM}$
$\alpha_{3,11}$	S_{NH}	$iN_{,BM}^{ALG}$	$gN/gCOD_{BM}$
$\alpha_{3,15}$	S_{PO4}	$iP_{,BM}^{ALG}$	$gP/gCOD_{BM}$
$\alpha_{3,16}$	S_{O2}	$iO_{,BM}^{ALG}-(32/12)\ iC_{,BM}^{ALG}+(24/14)iN_{,BM}^{ALG}$ $-(40/31)\ iP_{,BM}^{ALG}-(8)\ iH_{,BM}^{ALG}$	$gO_2/gCOD_{BM}$
$\alpha_{3,17}$	S_{H2O}	0.0404	$gH/gCOD_{BM}$
ρ_4 – Decay of X_{ALG}			
$\alpha_{4,1}$	X_{ALG}	-1	$gCOD_{BM}$ / $gCOD_{BM}$
$\alpha_{4,5}$	X_S	$(1-f_{XI,ALG})$	$gCOD_{XS}$ / $gCOD_{BM}$
$\alpha_{4,6}$	X_I	$f_{XI,ALG}$	
$\alpha_{4,9}$	S_{IC}	$iC_{BM}^{ALG}-(1- f_{XI,ALG})*iC_{XS}- f_{XI,ALG} *iC_{XI}$	$gC/gCOD_{BM}$
$\alpha_{4,11}$	S_{NH}	$iN_{BM}^{ALG}-(1- f_{XI,ALG})*iN_{XS}- f_{XI,ALG} *iN_{XI}$	$gN/gCOD_{BM}$
$\alpha_{4,15}$	S_{PO4}	$iP_{BM}^{ALG}-(1- f_{XI,ALG})*iP_{XS}- f_{XI,ALG} *iP_{XI}$	$gP/gCOD_{BM}$
ρ_5 – Aerobic growth of X_H on NH_4^+			
$\alpha_{5,3}$	X_H	1	$gCOD_{BM}$ / $gCOD_{BM}$
$\alpha_{5,7}$	S_S	$-1/Y_H$	$gCOD_{SS}$ / $gCOD_{BM}$
$\alpha_{5,9}$	S_{IC}	iC_{SS}/Y_H-iC_{BM}	gC/ $gCOD_{BM}$
$\alpha_{5,11}$	S_{NH}	iN_{SS}/Y_H-iN_{BM}	gN/ $gCOD_{BM}$
$\alpha_{5,15}$	S_{PO4}	iP_{SS}/Y_H-iP_{BM}	gP/ $gCOD_{BM}$
$\alpha_{5,16}$	S_{O2}	$-(1/Y_H-1)$	gO_2/ $gCOD_{BM}$
ρ_6 – Aerobic growth of X_H on NO_3^-			
$\alpha_{6,4}$	X_H	1	$gCOD_{BM}$ / $gCOD_{BM}$
$\alpha_{6,7}$	S_S	$-1/Y_H$	$gCOD_{SS}$ / $gCOD_{BM}$

$\alpha_{6,9}$	S_{IC}	$iC_{SS}/Y_H - iC_{BM}$	$gC/ gCOD_{BM}$
$\alpha_{6,13}$	S_{NO3}	$iN_{SS}/Y_H - iN_{BM}$	$gN/ gCOD_{BM}$
$\alpha_{6,15}$	S_{PO4}	$iP_{SS}/Y_H - iP_{BM}$	$gP/ gCOD_{BM}$
$\alpha_{6,16}$	S_{O2}	$-(1/Y_H - 1) - 64/14*(iN_{SS}/Y_H - iN_{BM})$	$gO_2/ gCOD_{BM}$
ρ_7 – Aerobic respiration of X_H			
$\alpha_{7,4}$	X_H	-1	$gCOD_{BM}$ / $gCOD_{BM}$
$\alpha_{7,9}$	S_{IC}	iC_{BM}	$gC/ gCOD_{BM}$
$\alpha_{7,11}$	S_{NH}	iN_{BM}	$gN/ gCOD_{BM}$
$\alpha_{7,15}$	S_{PO4}	iP_{BM}	$gP/ gCOD_{BM}$
$\alpha_{7,16}$	S_{O2}	-1	$gO_2/ gCOD_{BM}$
ρ_8 – Anoxic growth of X_H on NO_3^-			
$\alpha_{8,4}$	X_H	1	$gCOD_{BM}$ / $gCOD_{BM}$
$\alpha_{8,7}$	S_S	$-1/Y_{HNO3}$	$gCOD_{SS}/gCOD_{BM}$
$\alpha_{8,9}$	S_{IC}	$iC_{SS}/Y_{HNO3}-iC_{BM}$	$gC/gCOD_{BM}$
$\alpha_{8,11}$	S_{NH}	$iN_{SS}/Y_{HNO3}-iN_{BM}$	$gN/gCOD_{BM}$
$\alpha_{8,13}$	S_{NO3}	$-28/80*(1/Y_{HNO3}-1)$	$gN/gCOD_{BM}$
$\alpha_{8,14}$	S_{N2}	$28/80*(1/Y_{HNO3}-1)$	$gN/gCOD_{BM}$
$\alpha_{8,15}$	S_{PO4}	$iP_{SS}/Y_{HNO3}-iP_{BM}$	$gP/gCOD_{BM}$
ρ_9 – Anoxic growth of X_H on NO_2^-			
$\alpha_{9,4}$	X_H	1	$gCOD_{BM}$ / $gCOD_{BM}$
$\alpha_{9,7}$	S_S	$-1/Y_{HNO2}$	$gCOD_{SS}/gCOD_{BM}$
$\alpha_{9,9}$	S_{IC}	$iC_{SS}/Y_{HNO2}-iC_{BM}$	$gC/gCOD_{BM}$
$\alpha_{9,11}$	S_{NH}	$iN_{SS}/Y_{HNO2}-iN_{BM}$	$gN/gCOD_{BM}$
$\alpha_{9,12}$	S_{NO2}	$-28/48*(1/Y_{HNO2}-1)$	$gN/gCOD_{BM}$
$\alpha_{9,14}$	S_{N2}	$28/48*(1/Y_{HNO2}-1)$	$gN/gCOD_{BM}$
$\alpha_{9,15}$	S_{PO4}	$iP_{SS}/Y_{HNO2}-iP_{BM}$	$gP/gCOD_{BM}$
ρ_{10}– Anoxic respiration of X_H on NO_2^- and NO_3^-			
$\alpha_{10,4}$	X_H	-1	$gCOD_{BM}$ / $gCOD_{BM}$
$\alpha_{10,9}$	S_{IC}	iC_{BM}	$gC/gCOD_{BM}$
$\alpha_{10,11}$	S_{NH}	iN_{BM}	$gN/gCOD_{BM}$
$\alpha_{10,12}$	S_{NO2}	-14/64	$gN/gCOD_{BM}$
$\alpha_{10,13}$	S_{NO3}	-14/64	$gN/gCOD_{BM}$
$\alpha_{10,14}$	S_{N2}	28/64	$gN/gCOD_{BM}$
$\alpha_{10,15}$	S_{PO4}	iP_{BM}	$gP/ gCOD_{BM}$
ρ_{11}– Hydrolysis of slowly biodegradable COD			
$\alpha_{11,5}$	X_S	1	$gCOD_{XS}/ gCOD_{XS}$
$\alpha_{11,7}$	S_S	$1-f_{SI}$	$gCOD_{SS}$ / $gCOD_{XS}$
$\alpha_{11,8}$	S_I	f_{SI}	$gCOD_{SI}$ / $gCOD_{XS}$
$\alpha_{11,9}$	S_{IC}	$iC_{XS}-(1-f_{SI})*iC_{SS}-f_{SI}*iC_{SI}$	$gC/gCOD_{XS}$
$\alpha_{11,11}$	S_{NH}	$iN_{XS}-(1-f_{SI})*iN_{SS}-f_{SI}*iN_{SI}$	$gN/gCOD_{XS}$
$\alpha_{11,15}$	S_{PO4}	$iP_{XS}-(1-f_{SI})*iP_{SS}-f_{SI}*iP_{SI}$	$gP/gCOD_{XS}$
ρ_{12}– Hydrolysis of urea			
$\alpha_{12,9}$	S_{IC}	iC_{ND}	gC/gN_{urea}
$\alpha_{12,10}$	S_{ND}	-1	gN_{urea} / gN_{urea}
$\alpha_{12,11}$	S_{NH}	1	$gN_{ammonia}$ / gN_{urea}
$\alpha_{12,17}$	S_{H2O}	iH_{ND}	gH/ gN_{urea}
ρ_{13}– Decay of X_H			

$\alpha_{13,4}$	X_H	-1	$gCOD_{BM}$ / $gCOD_{BM}$
$\alpha_{13,5}$	X_S	$1-f_{XI}$	$gCOD_{,XS}/gCOD_{BM}$
$\alpha_{13,6}$	X_I	f_{XI}	$gCOD_{,XI}/gCOD_{BM}$
$\alpha_{13,9}$	S_{IC}	$iC_{BM}-(1-f_{XI})*iC_{XS}-f_{XI}*iC_{XI}$	$gC/gCOD_{BM}$
$\alpha_{13,11}$	S_{NH}	$iN_{BM}-(1-f_{XI})*iN_{XS}-f_{XI}*iN_{XI}$	$gN/gCOD_{BM}$
$\alpha_{13,15}$	S_{PO4}	$iP_{BM}-(1-f_{XI})*iP_{XS}-f_{XI}*iP_{XI}$	$gP/ gCOD_{BM}$
ρ_{14}– Aerobic growth of X_{AOB} on NH_4^+			
$\alpha_{14,2}$	X_{AOB}	1	$gCOD_{BM}$ / $gCOD_{BM}$
$\alpha_{14,9}$	S_{IC}	$-iC_{BM}$	$gC/ gCOD_{BM}$
$\alpha_{14,11}$	S_{NH}	$-iN_{BM}-1/Y_{AOB}$	$gN/ gCOD_{BM}$
$\alpha_{14,12}$	S_{NO2}	$1/Y_{AOB}$	$gN/ gCOD_{BM}$
$\alpha_{14,15}$	S_{PO4}	$-iP_{BM}$	$gP/ gCOD_{BM}$
$\alpha_{14,16}$	S_{O2}	$1-48/14*1/Y_{AOB}$	$gO_2/ gCOD_{BM}$
ρ_{15}– Aerobic respiration of X_{AOB}			
$\alpha_{15,2}$	X_{AOB}	-1	$gCOD_{BM}$ / $gCOD_{BM}$
$\alpha_{15,9}$	S_{IC}	iC_{BM}	$gC/ gCOD_{BM}$
$\alpha_{15,,11}$	S_{NH}	iN_{BM}	$gN/ gCOD_{BM}$
$\alpha_{15,15}$	S_{PO4}	iP_{BM}	$gP/ gCOD_{BM}$
$\alpha_{15,16}$	S_{O2}	-1	$gO_2/ gCOD_{BM}$
ρ_{16}– Decay of X_{AOB}			
$\alpha_{16,2}$	X_{AOB}	-1	$gCOD_{BM}$ / $gCOD_{BM}$
$\alpha_{16,5}$	X_S	$1-f_{XI}$	$gCOD_{,XS}/gCOD_{BM}$
$\alpha_{16,,6}$	X_I	f_{XI}	$gCOD_{,XI}/gCOD_{BM}$
$\alpha_{16,9}$	S_{IC}	$iC_{BM}-(1-f_{XI})*iC_{XS}-f_{XI}*iC_{XI}$	$gC/gCOD_{BM}$
$\alpha_{16,11}$	S_{NH}	$iN_{BM}-(1-f_{XI})*iN_{XS}-f_{XI}*iN_{XI}$	$gN/gCOD_{BM}$
$\alpha_{16,15}$	S_{PO4}	$iP_{BM}-(1-f_{XI})*iP_{XS}-f_{XI}*iP_{XI}$	$gCOD_{,XS}/gCOD_{BM}$
ρ_{17}– Aerobic growth of X_{NOB} on NO_3^-			
$\alpha_{17,3}$	X_{NOB}	1	$gCOD_{BM}$ / $gCOD_{BM}$
$\alpha_{17,9}$	S_{IC}	$-iC_{BM}$	$gC/ gCOD_{BM}$
$\alpha_{17,,11}$	S_{NH}	$-iN_{BM}$	$gN/ gCOD_{BM}$
$\alpha_{17,,12}$	S_{NO2}	$-1/Y_{NOB}$	$gN/ gCOD_{BM}$
$\alpha_{17,,13}$	S_{NO3}	$1/Y_{NOB}$	$gN/ gCOD_{BM}$
$\alpha_{17,,15}$	S_{PO4}	$-iP_{BM}$	$gP/ gCOD_{BM}$
$\alpha_{17,,16}$	S_{O2}	$1-16/14*1/Y_{NOB}$	$gO_2/ gCOD_{BM}$
ρ_{18}– Aerobic respiration of X_{NOB}			
$\alpha_{18,3}$	X_{NOB}	-1	$gCOD_{BM}$ / $gCOD_{BM}$
$\alpha_{18,9}$	S_{IC}	iC_{BM}	$gC/ gCOD_{BM}$
$\alpha_{18,,11}$	S_{NH}	iN_{BM}	$gN/ gCOD_{BM}$
$\alpha_{18,,15}$	S_{PO4}	iP_{BM}	$gP/ gCOD_{BM}$
$\alpha_{18,,16}$	S_{O2}	-1	$gO_2/ gCOD_{BM}$
ρ_{19}– Decay of X_{NOB}			
$\alpha_{19,3}$	X_{NOB}	-1	$gCOD_{BM}$ / $gCOD_{BM}$
$\alpha_{19,5}$	X_S	$1-f_{XI}$	$gCOD_{,XS}/gCOD_{BM}$
$\alpha_{19,,6}$	X_I	f_{XI}	$gCOD_{,XI}/gCOD_{BM}$
$\alpha_{19,,9}$	S_{IC}	$iC_{BM}-(1-f_{XI})*iC_{XS}-f_{XI}*iC_{XI}$	$gC/gCOD_{BM}$
$\alpha_{19,,11}$	S_{NH}	$iN_{BM}-(1-f_{XI})*iN_{XS}-f_{XI}*iN_{XI}$	$gN/gCOD_{BM}$
$\alpha_{19,15}$	S_{PO4}	$iP_{BM}-(1-f_{XI})*iP_{XS}-f_{XI}*iP_{XI}$	$gP/gCOD_{BM}$

ρ_{20} – Dissolution of O2			
$\alpha_{20,15}$	S_{O2}	1	[-]
ρ_{21} – Dissolution of CO_2			
$\alpha_{21,9}$	S_{IC}	1	[-]
ρ_{22} – Dissolution of NH_3			
$\alpha_{22,11}$	S_{NH}	1	[-]

Table SI.11.4. Kinetic parameter values in the ALBA model (Casagli et al.[1]).

Symbol	Description	Value ± std	Unit	Source
		Kinetic parameters		
		Biological model		
$\mu_{max,g,ALG}$	Maximum specific growth rate of X_{ALG}	2.5 ± 0.055	d^{-1}	1
$b_{max,r,ALG}$	Specific respiration rate of X_{ALG}	0.1	d^{-1}	1
$b_{max,d,ALG}$	Specific decay rate of X_{ALG}	0.03	d^{-1}	6
$\mu_{max,g,H}$	Maximum specific growth rate of X_H	6	d^{-1}	4
$b_{max,r,H}$	Specific aerobic respiration rate of X_H	0.3	d^{-1}	5
μ_{Hyd}	Hydrolysis rate of slowly biodegradable COD (X_S)	3	d^{-1}	6
μ_a	Hydrolysis rate of urea (SND)	0.25	d^{-1}	1
$b_{max,d,H}$	Specific decay rate of X_H	0.9	d^{-1}	1
$\mu_{max,g,AOB}$	Maximum specific aerobic growth rate of X_{AOB}	0.72 ±0.0005	d^{-1}	1
$b_{max,r,AOB}$	Specific aerobic respiration rate of X_{AOB}	0.05	d^{-1}	6
$b_{max,d,AOB}$	Specific decay rate of X_{AOB}	0.1	d^{-1}	8
$\mu_{max,g,NOB}$	Maximum specific aerobic growth rate of X_{NOB}	0.65 ±0.023	d^{-1}	1
$b_{max,r,NOB}$	Specific aerobic respiration rate of X_{NOB}	0.03	d^{-1}	5
$b_{max,d,NOB}$	Specific decay rate of X_{NOB}	0.08	d^{-1}	1
$K_{C,ALG}$	Inorganic carbon half-saturation constant for X_{ALG}	0.004	gCm^{-3}	8
$K_{O,ALG}$	Oxygen half-saturation constant for X_{ALG}	0.2	gO_2m^{-3}	5
$K_{N,ALG}$	Ammoniacal nitrogen half-saturation constant for X_{ALG}	0.1	gNm^{-3}	8
$K_{NO3,ALG}$	Nitrate half-saturation constant for X_{ALG}	0.3	gNm^{-3}	9
$K_{P,ALG}$	Phosphorus half-saturation constant for X_{ALG}	0.02	gNm^{-3}	9
$Ec_{50,O2}$	Oxygen value associated to 50% algae growth reduction	20	gO_2m^{-3}	1
n	Shape parameter associated to the Hill model	15	[-]	1
$K_{S,H}$	Soluble organic matter half-saturation constant for X_H	4	$gCODm^{-3}$	10
$K_{O,H}$	Oxygen half-saturation constant for X_H	0.2	gO_2m^{-3}	4
$K_{N,H}$	Ammonium half-saturation constant for X_H	0.05	gNm^{-3}	4
$K_{NO2,H}$	Nitrite half-saturation constant for X_H	0.2	gNm^{-3}	5
$K_{NO3,H}$	Nitrate half-saturation constant for X_H	0.5	gNm^{-3}	5
$K_{P,H}$	Phosphorus half-saturation constant for X_H	0.01	gPm^{-3}	4
K_{HYD}	Half saturation constant for hydrolysis	1	$gCODgCOD^{-1}$	5
$K_{C,AOB}$	Inorganic carbon half-saturation constant for X_{AOB}	0.5	gCm^{-3}	4
$K_{O,AOB}$	Oxygen half-saturation constant for AOB	0.8	gO_2m^{-3}	4
$K_{N,AOB}$	Ammonium half-saturation constant for X_{AOB}	0.5	gNm^{-3}	5
$K_{P,AOB}$	Phosphorus half-saturation constant for X_{AOB}	0.01	gPm^{-3}	4
$K_{C,NOB}$	Inorganic carbon half-saturation constant for X_{NOB}	0.5	gCm^{-3}	4

$K_{O,NOB}$	Oxygen half-saturation constant for X_{NOB}	2.2	gO_2m^{-3}	11
$K_{NO2,NOB}$	Nitrite half-saturation constant for X_{NOB}	0.5	gNm^{-3}	5
$K_{P,NOB}$	Phosphorus half-saturation constant for X_{NOB}	0.01	gPm^{-3}	4
η_{ANOX}	Efficiency reduction factor for denitrification process	0.6	[-]	12
I_{OPT}	Optimal irradiance for X_{ALG}	300 ± 3.814	$\mu mol\ m^{-2}\ s^{-1}$	13
α	Initial slope of irradiance response curve	0.01±0.0003	$\mu mol^{-1}\ m^2\ s^1$	1
ε	Light extinction coefficient	0.067	m^2 g COD^1	1
θ	Coefficient for temperature dependence for mass transfer	1.024	$°C^{-1}$	14
θ_H	Temperature coefficient for X_H decay	1.07	$°C^{-1}$	4
θ_{AOB}	Temperature coefficient for X_{AOB} decay	1.1	$°C^{-1}$	15
θ_{NOB}	Temperature coefficient for X_{NOB} decay	1.04	$°C^{-1}$	15
θ_{ALG}	Temperature coefficient for X_{ALG} decay	1.04	$°C^{-1}$	5
θ_{HYD}	Temperature coefficient for hydrolisis	1.04 ± 0.005	$°C^{-1}$	1
θ_{AMM}	Temperature coefficient for ammonification	1.12 ± 0.002	$°C^{-1}$	1
T_{max}^{ALG}	Maximum temperature threshold for X_{ALG}	42 ± 0.513	°C	1
T_{opt}^{ALG}	Optimal temperature for X_{ALG} growth	20 ± 0.148	°C	1
T_{min}^{ALG}	Minimum temperature threshold for X_{ALG}	-10 ± 1.524	°C	1
T_{max}^{AOB}	Maximum temperature threshold for X_{AOB}	40 ± 0.817	°C	1
T_{opt}^{AOB}	Optimal temperature for X_{AOB} growth	24.5 ± 0.232	°C	1
T_{min}^{AOB}	Minimum temperature threshold for X_{AOB}	-8 ± 0.741	°C	1
T_{max}^{NOB}	Maximum temperature threshold for X_{NOB}	38.5 ± 6.090	°C	1
T_{opt}^{NOB}	Optimal temperature for X_{NOB} growth	20 ± 0.940	°C	1
T_{min}^{NOB}	Minimum temperature threshold for X_{NOB}	-8 ±9.734	°C	1
T_{max}^{H}	Maximum temperature threshold for X_H	42 ± 1.919	°C	1
T_{opt}^{H}	Optimal temperature for X_H growth	25 ± 0.634	°C	1
T_{min}^{H}	Minimum temperature threshold for X_H	-3 ± 0.335	°C	1
pH_{max}^{ALG}	Maximum pH threshold for X_{ALG}	12 ± 0.039	-	1
pH_{opt}^{ALG}	Optimal pH for X_{ALG} growth	8.4 ± 0.066	-	1
pH_{min}^{ALG}	Minimum pH threshold for X_{ALG}	2 ± 0.562	-	1
pH_{max}^{AOB}	Maximum pH threshold for X_{AOB}	12.4 ± 0.115	-	1
pH_{opt}^{AOB}	Optimal pH for X_{AOB} growth	8.1 ±0.078	-	1
pH_{min}^{AOB}	Minimum pH threshold for X_{AOB}	5.8 ± 0.355	-	1
pH_{max}^{NOB}	Maximum pH threshold for X_{NOB}	12.1 ± 0.463	-	1
pH_{opt}^{NOB}	Optimal pH for X_{NOB} growth	7.9 ± 0.320	-	1
pH_{min}^{NOB}	Minimum pH threshold for X_{NOB}	5 ±0.568	-	1
pH_{max}^{H}	Maximum pH threshold for X_H	11.5 ±0.022	-	1
pH_{opt}^{H}	Optimal pH for X_H growth	7 ± 0.066	-	1
pH_{min}^{H}	Maximum pH threshold for X_{ALG}	2 ±0.344	-	1

		**Gas-liquid exchange with atmosphere**		
K_La	Mass transfer coefficient for O_2	34 ± 0.1	d^{-1}	1
H_{O2}	Henry's constant for O_2	Eq(SI6.3)	$gO_2\ atm^{-1}m^{-3}$	16
H_{CO2}	Henry's constant for carbon CO_2	Eq(SI6.4)	$gCO_2\ atm^{-1}m^{-3}$	16
H_{NH3}	Henry's constant for NH_3	Eq(SI6.5)	$gNH_3\ atm^{-1}m^{-3}$	16
D_{O2}	Mass diffusion coefficient for O_2	2.5e-009	m^2s^{-1}	17
D_{CO2}	Mass diffusion coefficient for CO_2	2.1e-009	m^2s^{-1}	17
D_{NH3}	Mass diffusion coefficient for NH_3	2.4e-009	m^2s^{-1}	17
p_{O2}	Partial pressure of O_2 in gas phase	0.21	atm	1
p_{CO2}	Partial pressure of CO_2 in gas phase	0.0004	atm	1
p_{NH3}	Partial pressure of NH_3 in gas phase	1.5e-006	atm	1
		**pH sub-model**		
pka_{CO2}	Acid dissociation constant for carbonic acid - bicarbonate balance	6.37	-	7
pka_{HCO3^-}	Acid dissociation constant for carbonic acid - bicarbonate balance	10.33	-	7
pka_{NH4^+}	Acid dissociation constant for bicarbonate-carbonate balance	9.25	-	7
pka_{HNO2}	Acid dissociation constant for ammonia-ammonium balance	3.35	-	7
pka_{HNO3}	Acid dissociation constant for nitrous acid -nitrite balance	-1.64	-	7
pka_{H3PO4}	Acid dissociation constant for nitric acid-nitrate balance	2.14	-	7
pka_{H2PO4^-}	Acid dissociation constant for phosphoric acid-dihydrogen phosphate balance	7.21	-	7
$pka_{HPO4^{2-}}$	Acid dissociation constant for dihydrogen phosphate-hydrogen phosphate balance	12.67	-	7

Table SI.11.5. pH sub-model equation system in the ALBA model (Casagli et al.[1]).

Description	Expression [mol m^{-3}]	K_A (293 K) [M]
1- Mass balance	$\frac{S_{NH}}{14} = NH_3+NH_4^+$	
2 - Dissociation $NH_4^+ \leftrightarrow NH_3+H^+$	$NH_4^+ = \left(\frac{S_{NH}/14}{1+\frac{(Ka_{NH4}\cdot 10^3)}{H^+}}\right)$	$K_{A,NH4}$: 5.62E-10
3- Mass balance	$\frac{S_{NO_2}}{14} = NO_2^-+HNO_2$	
4 - Dissociation $HNO_2 \leftrightarrow NO_2^-+H^+$	$HNO_2^- = \left(\frac{S_{NO_2}/14}{1+\frac{(Ka_{NO_2}\cdot 10^3)}{H^+}}\right)$	$K_{A,HNO2}$: 4.47E-04
5- Mass balance	$\frac{S_{NO_3}}{14} = NO_3^-+HNO_3$	
6 - Dissociation $HNO_3 \leftrightarrow NO_3^-+H^+$	$HNO_3^- = \left(\frac{S_{NO_3}/14}{1+\frac{(Ka_{NO_3}\cdot 10^3)}{H^+}}\right)$	$K_{A,HNO3}$: 4.37E+01
7- Mass balance	$\frac{S_{IC}}{12} = CO_2+HCO_3^-+CO_3^{2-}$	
8 - Dissociation $H_2O+CO_2 \leftrightarrow HCO_3^-+H^+$	$CO_2 = \frac{S_{IC}/12}{1+\frac{(Ka_{CO2}*10^3)}{H^+}+\frac{(Ka_{CO2}*K_{a_{HCO3}}*10^6)}{(H^+)^2}}$	$K_{A,H2CO3}$: 4.27E-07
9 - Dissociation $HCO_3^- \leftrightarrow CO_3^{2-}+H^+$	$HCO_3^- = \left(\frac{S_{IC}/12}{1+\frac{H^+}{(K_{a_{CO2}}*10^3)}+\frac{(Ka_{HCO3}*10^3)}{H^+}}\right)$	$K_{A,HCO3}$: 4.68E-11
10- Mass balance	$\frac{S_{PO4}}{31} = H_3PO_4+H_2PO_4^-+HPO_4^{2-}+PO_4^{3-}$	
11 - Dissociation $H_3PO_4 \leftrightarrow H_2PO_4^-+H^+$	$H_3PO4 = \frac{S_{PO_4}/31}{1+\frac{(Ka_{H3PO4}*10^3)}{H^+}+\frac{(Ka_{H3PO4}*K_{aH2PO4}*10^6)}{(H^+)^2}+\frac{(Ka_{H3PO4}*K_{aH2PO4}*K_{aHPO4}*10^9)}{(H^+)^3}}$	$K_{A,H3PO4}$: 7.24E-03
12 - Dissociation $H_2PO_4^- \leftrightarrow HPO_4^{2-}+H^+$	$H_2PO_4^- = \frac{S_{PO_4}/31}{1+\frac{H^+}{(K_{A_{H3PO4}}*10^3)}+\frac{(Ka_{H2PO4}*10^3)}{H^+}+\frac{(Ka_{H2PO4}*K_{aHPO4}*10^6)}{(H^+)^2}}$	$K_{A,H2PO4}$: 6.17E-08

13 - Dissociation $HPO_4^{2-} \leftrightarrow PO_4^{3-}+H^+$	$HPO_4^{2-} = \frac{S_{PO_4}/31}{1+\frac{(H^+)^2}{(K_{A_{H3PO4}}*K_{a_{H2PO4}}*10^6)}+\frac{H^+}{(K_{a_{H2PO4}}*10^3)}+\frac{(Ka_{HPO4}*10^3)}{H^+}}$	$K_{A,HPO4}$: 2.14E-13
14 - Dissociation $H_2O \leftrightarrow OH^-+H^+$	$OH^- = \frac{Ka_w*10^3}{H^+}$	$K_{A,w}$: 1.00E-14
15 – Charge balance	$H^+ + NH_4^+ + \Delta_{CAT,AN} - OH^- - NO_2^- - NO_3^- - HCO_3^- - 2CO_3^{2-} - H_2PO_4^- - 2HPO_4^{2-} - 3PO_4^{3-} = 0$	-

The temperature influence on the dissociation constants (k_a) was considered by using the van't Hoff equation:

$$\ln\left(\frac{K_{a,\,T}}{K_{a,\,Tref}}\right) = \frac{\Delta H^{\circ}}{R}\cdot\left(\frac{1}{T_{ref}}-\frac{1}{T+273.15}\right) \qquad \text{(SI.11.1)}$$

In Equation SI6.4, T_{ref} is the standard temperature (298.15 K) for which the equilibrium coefficient value ($K_{a,Tref}$, [mol L^{-1}]) is known, T is the temperature at which evaluate the equilibrium coefficient value ($K_{a,T}$, [mol L^{-1}]), R is the gas law constant [J K^{-1} mol^{-1}] and ΔH° is the heat of reaction at standard temperature and pressure [J].

Table SI.11.6. Biological process rates in the ALBA model (Casagli et al.[1]).

Group				Process	Rate
Algae (X_{ALG})			ρ_1	Growth on S_{NH}	$\mu_{max,g,ALG}\cdot f_I\cdot f_{T_{ALG}}\cdot f_{pH_{ALG}}\cdot f_{O_2,g}\cdot \min\left(\frac{S_{IC}}{K_{C,ALG}+S_{IC}},\frac{S_{NH}}{K_{N,ALG}+S_{NH}},\frac{S_{PO4}}{K_{P,ALG}+S_{PO4}}\right)\cdot X_{ALG}$
			ρ_2	Growth on S_{NO3}	$\mu_{max,g,ALG}\cdot f_I\cdot f_{T_{ALG}}\cdot f_{pH_{ALG}}\cdot f_{O_2,g}\cdot\frac{K_{N,ALG}}{K_{N,ALG}+S_{NH}}\cdot \min\left(\frac{S_{IC}}{K_{C,ALG}+S_{IC}},\frac{S_{NO3}}{K_{NO3,ALG}+S_{NO3}},\frac{S_{PO4}}{K_{P,ALG}+S_{PO4}}\right)\cdot X_{ALG}$
			ρ_3	Aerobic respiration	$b_{max,r,ALG}\cdot f_{T_{ALG}}\cdot f_{pH_{ALG}}\cdot\frac{S_{O2}}{K_{O,ALG}+S_{O2}}\cdot X_{ALG}$
			ρ_4	Decay	$b_{max,d,ALG}\cdot(\theta_{ALG}^{(T-20)}\cdot f_{pH_{ALG}}+f_{O2,d})\cdot X_{ALG}$
Heterotrophic bacteria (X_H)		Aerobic	ρ_5	Growth on S_{NH}	$\mu_{max,g,H}\cdot f_{T_H}\cdot f_{pH_H}\cdot \min\left(\frac{S_S}{K_{S,H}+S_S},\frac{S_{O2}}{K_{O,H}+S_{O2}},\frac{S_{NH}}{K_{N,H}+S_{NH}},\frac{S_{PO4}}{K_{P,H}+S_{PO4}}\right)\cdot X_H$
			ρ_6	Growth on S_{NO3}	$\mu_{max,g,H}\cdot f_{T_H}\cdot f_{pH_H}\cdot\frac{K_{N,H}}{K_{N,H}+S_{NH}}\cdot\min\left(\frac{S_S}{K_{S,H}+S_S},\frac{S_{O2}}{K_{O,H}+S_{O2}},\frac{S_{NO3}}{K_{NO3,H}+S_{NO3}},\frac{S_{PO4}}{K_{P,H}+S_{PO4}}\right)\cdot X_H$
			ρ_7	Respiration	$b_{max,r,H}\cdot f_{T_H}\cdot f_{pH_H}\cdot\frac{S_{O2}}{K_{O,ALG}+S_{O2}}\cdot X_H$
		Anoxic	ρ_8	Growth on S_{NO2}	$\mu_{max,g,H}\cdot\eta_{ANOX}\cdot f_{T_H}\cdot f_{pH_H}\cdot\frac{K_{O,H}}{K_{O,H}+S_{O2}}\cdot\min\left(\frac{S_S}{K_{S,H}+S_S},\frac{S_{NO2}}{K_{NO2,H}+S_{NO2}},\frac{S_{PO4}}{K_{P,H}+S_{PO4}}\right)\cdot X_H$
			ρ_9	Growth on S_{NO3}	$\mu_{max,g,H}\cdot\eta_{ANOX}\cdot f_{T_H}\cdot f_{pH_H}\cdot\frac{K_{O,H}}{K_{O,H}+S_{O2}}\cdot\min\left(\frac{S_S}{K_{S,H}+S_S},\frac{S_{NO3}}{K_{NO3,H}+S_{NO3}},\frac{S_{PO4}}{K_{P,H}+S_{PO4}}\right)\cdot X_H$
			ρ_{10}	Respiration	$b_{max,r,H}\cdot\eta_{ANOX}\cdot f_{T_H}\cdot f_{pH_H}\cdot\frac{K_{O,H}}{K_{O,H}+S_{O2}}\cdot\min\left(\frac{S_{NO2}}{K_{NO2,H}+S_{NO2}},\frac{S_{NO3}}{K_{NO3,H}+S_{NO3}}\right)\cdot X_H$
			ρ_{11}	Hydrolysis	$\mu_{Hyd}\cdot\theta_{HYD}^{(T-20)}\cdot f_{pH_{Hyd}}\cdot\frac{X_S/X_H}{K_{HYD}+(X_S/X_H)}\cdot X_H$
			ρ_{12}	Ammonification	$\mu_a\cdot\theta_{AMM}^{(T-20)}\cdot f_{pH_a}\cdot\frac{S_{ND}}{K_a+S_{ND}}\cdot X_H$
			ρ_{13}	Decay	$b_{max,d,H}\cdot\theta_H^{(T-20)}\cdot f_{pH_H}\cdot X_H$
Nitrifying bacteria	X_{AOB}	Aerobic	ρ_{14}	Growth	$\mu_{max,g,AOB}\cdot f_{T_{AOB}}\cdot f_{pH_{AOB}}\cdot\min\left(\frac{S_{NH}}{K_{N,AOB}+S_{NH}},\frac{S_{O2}}{K_{O,AOB}+S_{O2}},\frac{S_{IC}}{K_{C,AOB}+S_{IC}},\frac{S_{PO4}}{K_{P,AOB}+S_{PO4}}\right)\cdot X_{AOB}$
			ρ_{15}	Respiration	$b_{max,r,AOB}\cdot f_{T_{AOB}}\cdot f_{pH_{AOB}}\cdot\frac{S_{O2}}{K_{O,AOB}+S_{O2}}\cdot X_{AOB}$
			ρ_{16}	Decay	$b_{max,d,AOB}\cdot\theta_{AOB}^{(T-20)}\cdot f_{pH_{AOB}}\cdot X_{AOB}$
	X_{NOB}	Aerobic	ρ_{17}	Growth	$\mu_{max,g,NOB}\cdot f_{T_{NOB}}\cdot f_{pH_{NOB}}\cdot\min\left(\frac{S_{NO2}}{K_{NO2,NOB}+S_{NO2}},\frac{S_{O2}}{K_{O,NOB}+S_{O2}},\frac{S_{IC}}{K_{C,NOB}+S_{IC}},\frac{S_{PO4}}{K_{P,NOB}+S_{PO4}}\right)\cdot X_{AOB}$
			ρ_{18}	Respiration	$b_{max,r,NOB}\cdot f_{T_{NOB}}\cdot f_{pH_{NOB}}\cdot\frac{S_{O2}}{K_{O,NOB}+S_{O2}}\cdot X_{NOB}$
			ρ_{19}	Decay	$b_{max,d,NOB}\cdot\theta_{NOB}^{(T-20)}\cdot f_{pH_{NOB}}\cdot X_{NOB}$

* S_{IC} in the Monod terms incudes the inorganic carbon from CO_2 and HCO^{3-}, without accounting for the contribution given by CO_3^{2-} . The concentration of CO_2 and HCO^{3-} is estimated using the pH sub-model, as shown in Table SI.11.5.

Table SI.11.7. Gas-liquid transfer rates in the ALBA model (Casagli et al.[1]).

Gas – liquid mass transfer		
**Process**	**Description**	**Unit**
ρ_{20} - oxygen stripping/dissolution	$\theta^{T-20} \cdot kLa \cdot (H_{O2}(T) \cdot p_{O2} - S_{O2})$	$gO_2\ m^{-3}\ d^{-1}$
ρ_{21} - carbon dioxide stripping/dissolution	$\theta^{T-20} \cdot kLa \cdot \left(\frac{D_{CO2}}{D_{O2}}\right)^{0.5} \cdot (H_{CO2}(T) \cdot p_{CO2} - CO_2)$	$gC\text{-}CO_2\ m^{-3}\ d^{-1}$
ρ_{22}- ammonia stripping	$\theta^{T-20} \cdot kLa \cdot \left(\frac{D_{NH3}}{D_{O2}}\right)^{0.5} \cdot (H_{NH3}(T) \cdot p_{NH3} - NH_3)$	$gN\text{-}NH_3\ m^{-3}\ d^{-1}$

SI.12 Experimental measurement of k_La

The volumetric mass transfer coefficient (k_La) was estimated according to the ASCE Standard[18]. In brief, the HRABP was filled with clean water, then the water volume was de-oxygenated using reagent-grade chemicals. To this purpose, sodium sulphite (Na_2SO_3) was uniformly distributed on the surface of the HRABP, to achieve water de-oxygenation. Then, the water re-oxygenation was monitored until reaching a value close to the DO saturation at the test temperature. DO data were measured using two DO probes (Hach-Lange, LDO101), each connected to a multi-meter data logger (Hach-Lange, HQ40D), recording DO data every 30 s. The DO was measured in two different points of the HRABP channels, i.e. one immediately after the paddlewheel and one at the opposite location of the loop, to account for different mass transfer rates in the two zones[19]. The overall mass transfer coefficient was then calculated by assuming the average value of the two determinations.

The DO data were then analysed to estimate the volumetric mass transfer coefficient (k_La), according to the following model:

$$C=C_{\infty}^{*}-\left(C_{\infty}^{*}-C_0\right)*\exp(-k_La*t)$$

Where: C is the DO concentration [g m^{-3}], C_{∞}^{*} is the steady-state DO saturation concentration as time approaches infinity [g m^{-3}], C_0 is the initial DO concentration at time zero [g m^{-3}], k_La is the volumetric mass transfer coefficient [d^{-1}] and t is the test time [d].

The test was carried out at approximately 20 °C. To refer to standard conditions, the obtained k_La value at the test temperature was, according to ASCE[18]:

$$k_La_{20}=k_La*\theta^{(20-T)}$$

Where: k_La_{20} is the volumetric mass transfer coefficient corrected to 20 °C and θ = 1.024 is the recommended empirical temperature correction factor [-].

Nonlinear regression was performed in MS Excel (GRG nonlinear solver), to estimate the k_La and C_∞^* values. The overall k_La_{20} was 30.6 ± 0.4 d^{-1}. The results of the re-oxygenation test are reported in Figure SI.12.1 and Table SI.12.1.

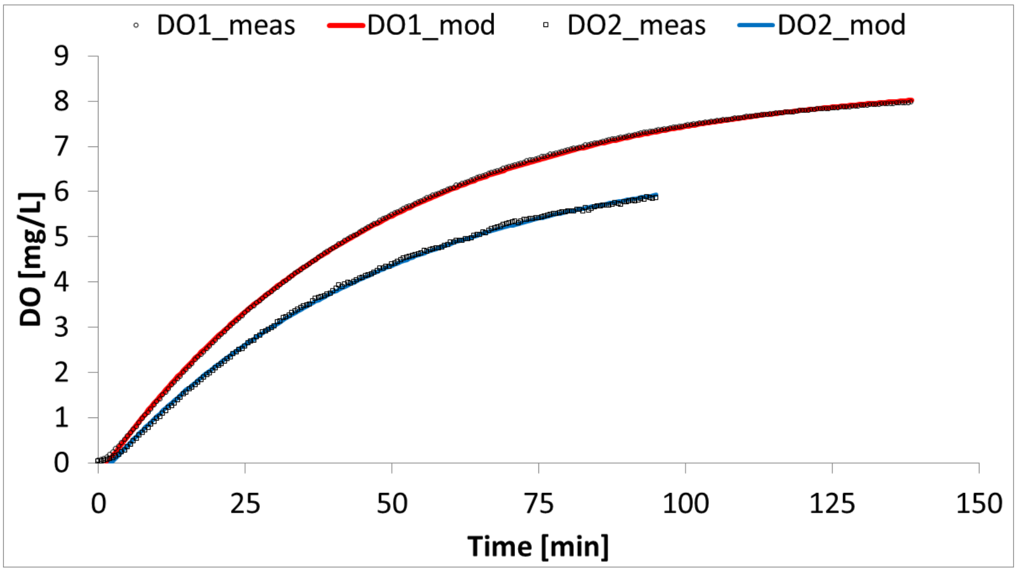

Figure SI.12.1. Measured and modelled DO data (DO_1: DO data measured immediately after the paddlewheel, DO_2: DO data measured on the opposite side).

Table. SI.12.1. Estimated k_La values for the HRABP (DO_1: DO data measured immediately after the paddlewheel, DO_2: DO data measured on the opposite side). The overall value is indicated as average ± standard deviation of the two measurements.

Parameter	Unit	DO_1	DO_2	Overall
Temperature	[°C]	20.0	19.3	-
k_La	[d-1]	30.9	29.9	30.4 ± 0.7
k_La_{20}	[d-1]	30.9	30.3	30.6 ± 0.4

SI.13 Parameter uncertainty and error propagation

Once the model was calibrated and validated, a dynamic sensitivity analysis was run, including all the experimental periods covered from the monitoring campaigns (i.e. 31/05/2016 – 06/12/2016), therefore using the actual environmental conditions (and not the average values, as shown in the manuscript in Figure 1). The sensitivity functions were then computed in these real conditions for all the most sensitive parameters investigated, again choosing the absolute-relative sensitivity function (equation SI.13.1).

The parameter standard deviation was then derived from the Fisher Information Matrix *F*. The Fisher analysis is based on the local sensitivity functions $\partial^{a,r}_{y_i,p_j}$, and turned out to be efficient for biological dynamic systems [20,21]. The *F* matrix (Eq. SI.13.3) was computed from the sensitivity matrix $\Delta\ Y_p$ (Equation SI.13.2) and the covariance matrix of measured standard deviations, *C*. The standard deviation δ_j associated to parameters p_j was then computed as reported in Eq. SI.13.4.

$$\partial^{a,r}_{y_i,p_j} = \frac{\partial y_i}{\partial p_j} \qquad \text{SI.13.1}$$

$$\Delta = \left[\frac{\partial y}{\partial p_1}, \ldots, \frac{\partial y}{\partial p_m}\right] \qquad \text{SI.13.2}$$

$$F = \sum_{k=1}^{K} \Delta^T C^{-1} \Delta \qquad \text{SI.13.3}$$

$$\delta_j^{\,2} = \left(F^{-1}\right)_{j,j} \qquad \text{SI.13.4}$$

After performing the sensitivity analysis, and estimating the parameter standard error, as described above, the error propagation σ_{y_i} of the model predictions for y_i was also computed (equation SI.13.5):

$$\sigma_{y_i}(t) = \sqrt{\sum_{j=1}^{m} \left(\frac{\partial y_i}{\partial p_j}(t)\right)^2 \sigma_{p_j}^2} \qquad \text{SI.13.5}$$

Where: p_j are the model parameters, σ_{p_j} their standard deviations, $y_i(p_1, \ldots, p_m)$ is the model solution for each predicted state y_i, at a given time t, and σ_{y_i} is the prediction standard deviation of the model result. Then, the 95% confidence intervals on the modelled predictions of relevant parameters (i.e., TSS, COD_s, X_{ALG}, S_{NH}, S_{NO2}, S_{NO3}, and S_{O2}) were estimated by the interval $[y_i\text{-}1.96\ \sigma_{y_i} \quad y_i+1.96\ \sigma_{y_i}]$ and are shown in the manuscript in Figure 2 and 3.

Table SI.13.1. Most sensible parameters identified from the sensitivity analysis performed (as briefly described in the manuscript, Section 2.2.3, and more in detail in Casagli et al.[1]); the nominal and calibrated values with their standard deviation (obtained with the procedure described above, Eq.SI.13.4).

Parameter/Symbol	Nominal value	Reference	Calibrated value	St.Dev.
Algae maximum specific growth rate [$\mu_{max,g,ALG}$]	1.5 d^{-1}	22	2.5 d^{-1}	0.019 d^{-1}
AOB maximum specific growth rate [$\mu_{max,g,AOB}$]	0.9 d^{-1}	6	0.72 d^{-1}	0.056 d^{-1}
NOB maximum specific growth [$\mu_{max,g,NOB}$]	0.67 d^{-1}	6	0.65 d^{-1}	0.002 d^{-1}
Light optimal value for growth [I_{OPT}]	275 μmol m^{-2} s^{-1}	13	300 μmol m^{-2} s^{-1}	6.270 μmol m^{-2} s^{-1}
Light extinction coefficient [ε]	0.067 ± 0.001 m^2 $gCOD^{-1}$	[measured]	-	0.005 m^2 $gCOD^{-1}$
Initial slope of PI curve [α]	0.027	13	0.01 μmol^{-1} m^2 $s^1 d^{-1}$	0.0008 μmol^{-1} m^2 $s^1 d^{-1}$
Mass transfer coefficient [k_La]	25 d^{-1}	9	34 d^{-1}	0.172 d^{-1}
Coefficient for temperature correction for hydrolysis [θ_{Hyd}]	1.07	5	1.04	0.007
Coefficient for temperature correction for ammonification [θ_{AMM}]	1.07	5	1.12	-

Parameter/Symbol	Nominal value			Reference	Calibrated value and St.Dev.					
	Min	Opt	Max		Min	St.Dev.	Opt	St.Dev.	Max	St.Dev.
Cardinal temperature values for X_{ALG} [CTMI]	1.1	32.5	39.3	23	-10	3.021	20	0.296	42	0.671
Cardinal temperature values for X_{AOB} [CTMI]	5	25-35	35	10	-8	2.249	24.5	0.382	40	1.174
Cardinal temperature values for X_{NOB} [CTMI]	5	25-30	37	10	-8	7.310	20	0.988	38.5	0.272
Cardinal temperature values for X_H [CTMI]	5	40	47	24	-3	5.091	25	1.450	42	6.257
Cardinal pH values for X_{ALG} [CPM]	2.24	7.34	10	25	2	1.240	8.4	0.428	12	0.826
Cardinal pH values for X_{AOB} [CPM]	5.8	7.8-8	9	10	5.8	0.029	8.1	0.095	12.3	0.724
Cardinal pH values for X_{NOB} [CPM]	6.5	7.6-8	8.6	10	5	0.053	7.9	0.031	12.1	1.644
Cardinal pH values for X_H [CPM]	4	7	9	26	2	7.598	7	0.984	11.5	0.446

Notes: The cardinal temperature values are expressed in °C; ± St.Dev. is the standard deviation computed for the calibrated parameters .

SI.14 Residual analysis results

A)

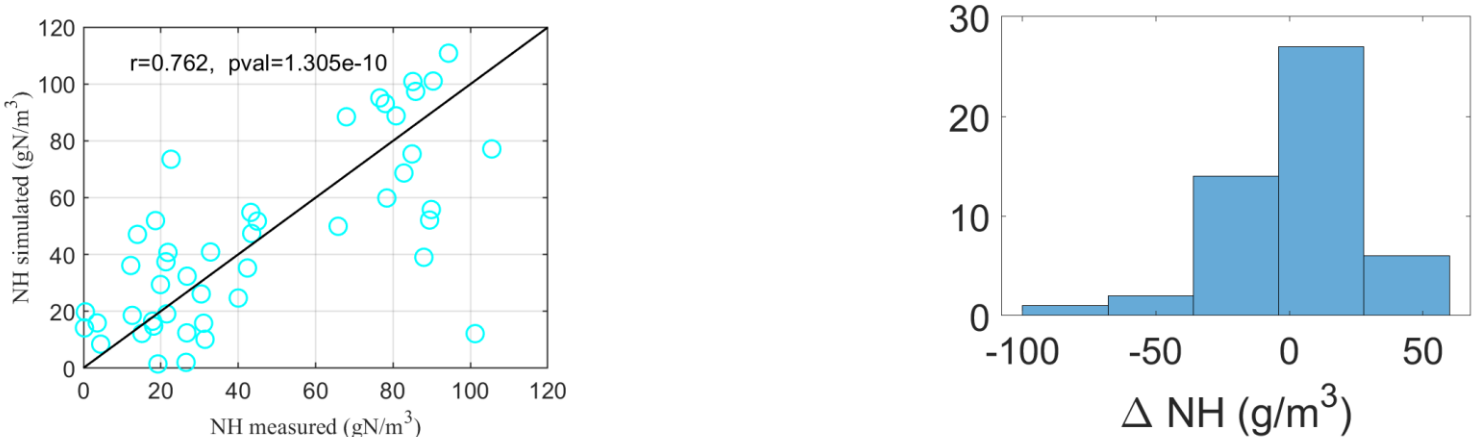

B)

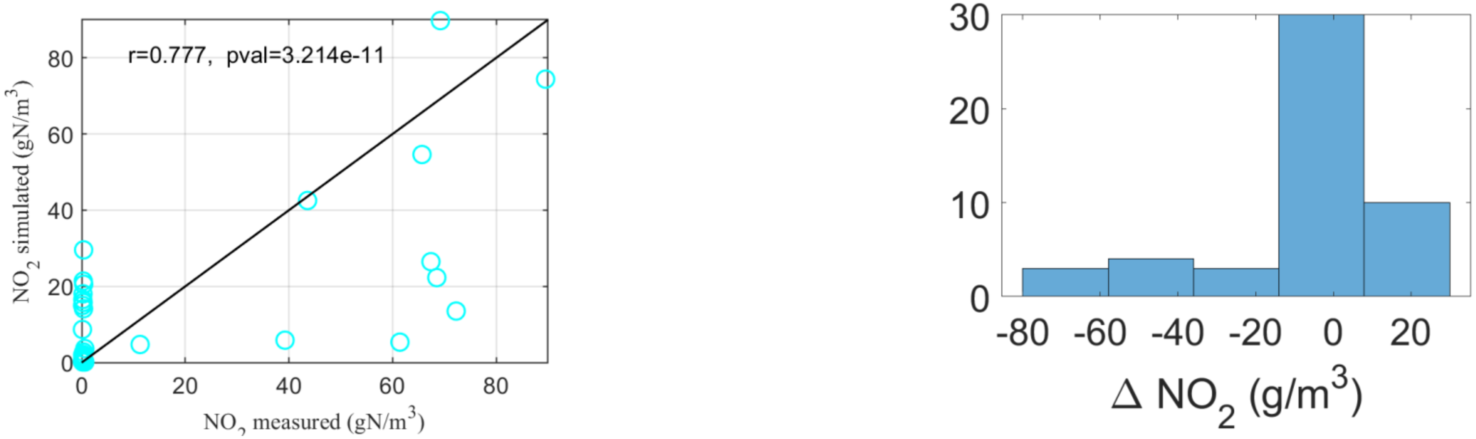

C)

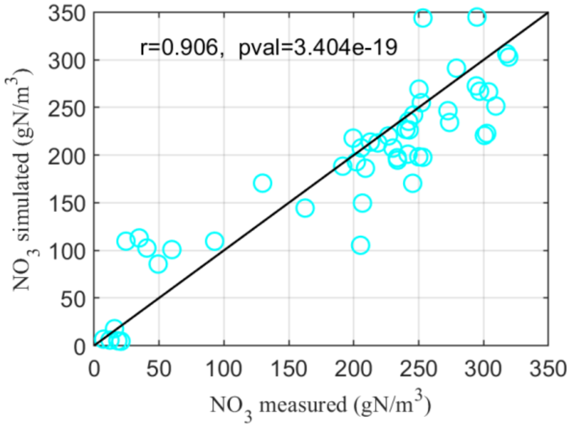
r=0.906, pval=3.404e-19
NO3 simulated (gN/m3)
NO3 measured (gN/m3)

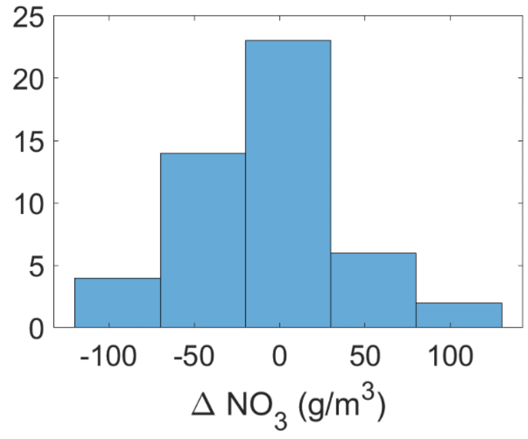
Δ NO3 (g/m3)

D)

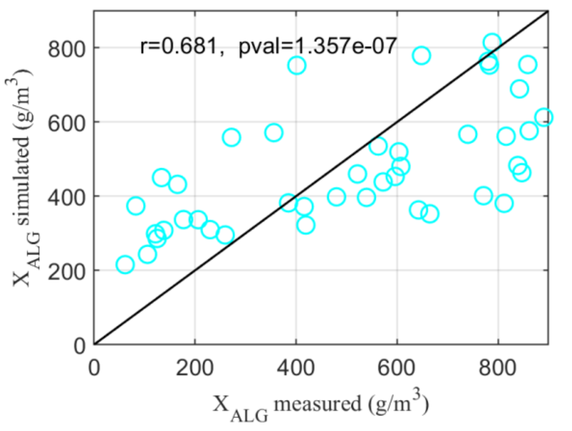
r=0.681, pval=1.357e-07
XALG simulated (g/m3)
XALG measured (g/m3)

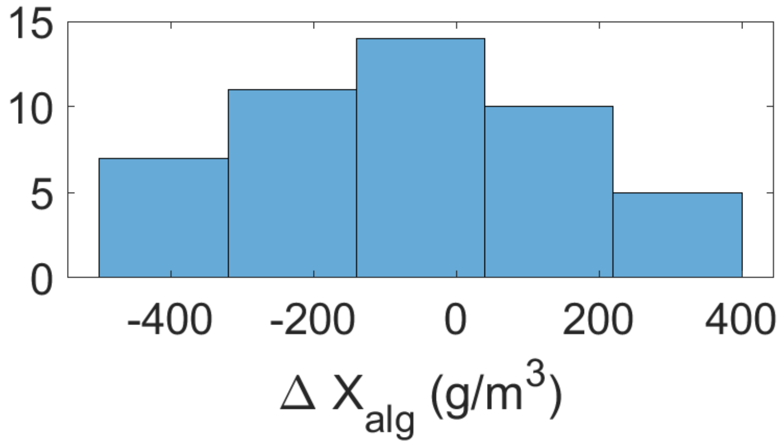
Δ Xalg (g/m3)

E)

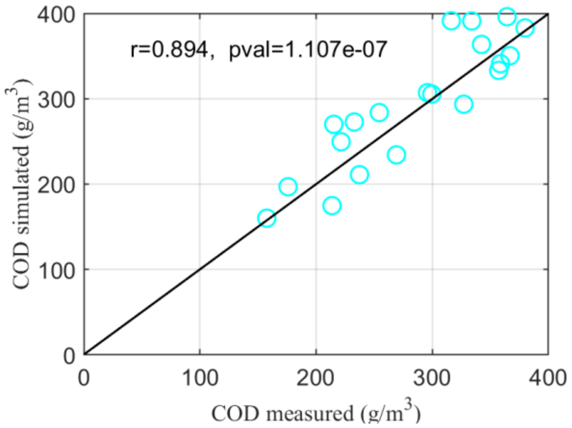
r=0.894, pval=1.107e-07
COD simulated (g/m^3)
COD measured (g/m^3)

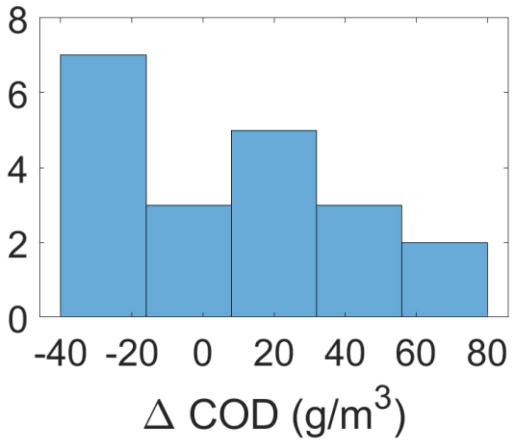
Δ COD (g/m^3)

F)

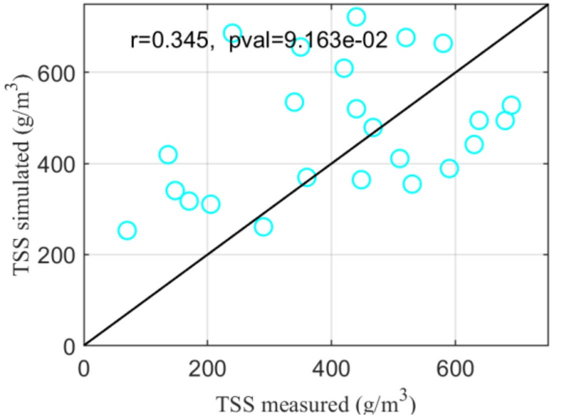
r=0.345, pval=9.163e-02
TSS simulated (g/m^3)
TSS measured (g/m^3)

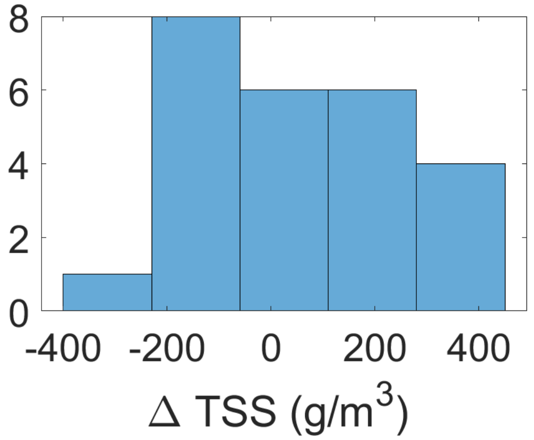
Δ TSS (g/m^3)

G)

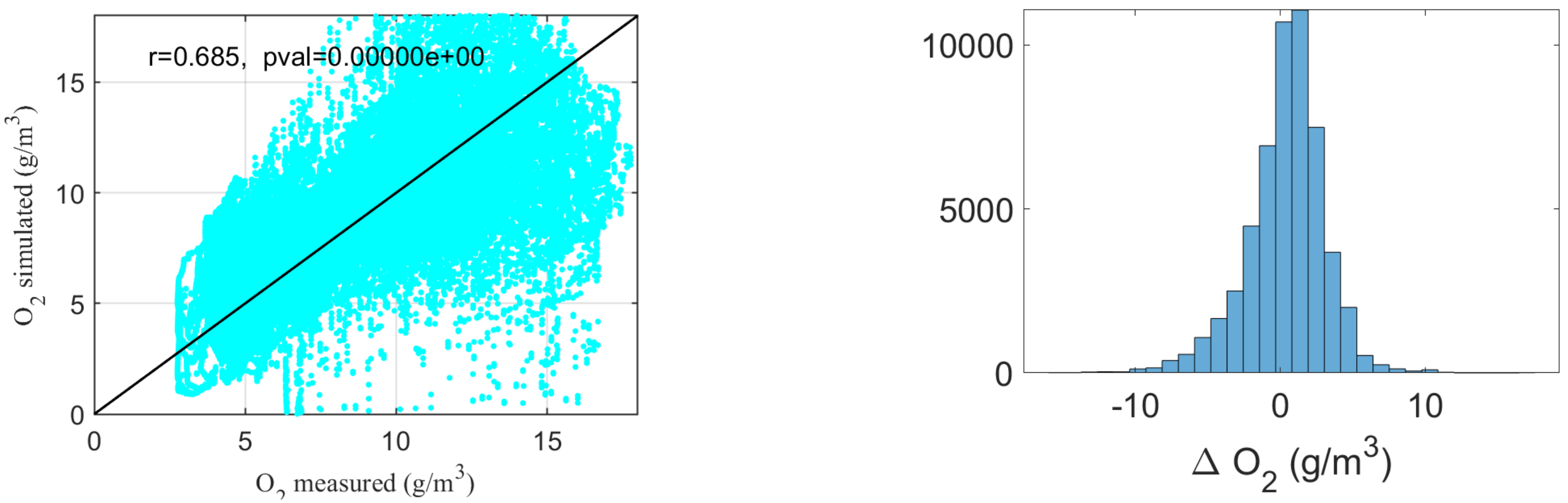

H)

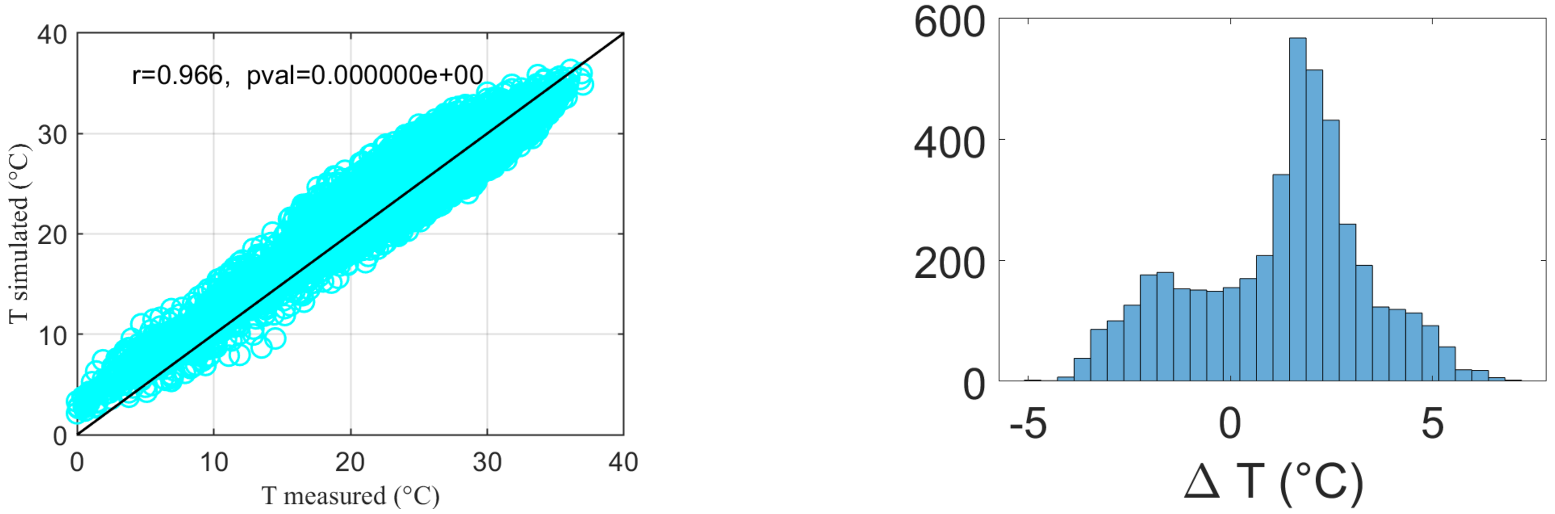

Figure SI.14.1. Simulated vs measured data (left) and residual analysis (right) for the monitored variables: A) ammonium [gN m^{-3}]; B) nitrite [gN m^{-3}]; C) nitrate [gN m^{-3}]; D) algal biomass [gCOD m^{-3}]; E) Soluble COD [gCOD m^{-3}]; F)Total Suspended Solids [gTSS m^{-3}]; G) Dissolved oxygen [gO_2 m^{-3}]; H) Temperature in the pond [°C]. pval is the p-value computed.

SI.15 Economic computation for alkalinity addition in the raceway

An economic analysis accounting for the price of soda to regulate alkalinity was performed. Alkalinity in the reactor was assumed to be increased through addition of NaOH, that has a unit cost of approximately 0.4 $\$$ kg^{-1}. Considering the molecular weight of NaOH (40 g mol^{-1}) and the additional alkalinity of 20 mol m^{-3} (obtained by increasing the influent alkalinity). The resulting alkalinity request is 0.8 kg(NaOH) m^{-3} leading to a cost for adding NaOH of 0.32 $\$$ m^{-3}.
From scenario analysis simulation results, the actual nitrogen removal rate (i.e. considering NH_3 stripped as untreated nitrogen) for Spring season (Tab SI.10.1), was considered. Specifically for the scenarios S4 (HRT 5 d, liquid depth 0.2, no alkalinity addition in the influent) and S11 (HRT 5 d, liquid depth 0.2, increased influent alkalinity), the actual nitrogen removal rate was 11.41 gN-TAN m^{-2} d^{-1} and 16.29 gN-TAN m^{-2} d^{-1} respectively, with a gain of 4.88 gN-TAN m^{-2} d^{-1} in S11, representing approximately a 30% increase in the nitrogen removal rate. Considering an operational cost for treating nitrogen of 6 $\$$ kgN^{-1} (Lin et al.[27]), it means that treating an additional 30% of nitrogen with additional alkalinity has a value estimated to be 0.03 $\$$ m^{-2} d^{-1}.
The cost of alkalinity addition is 0.0128 $\$$ m^{-2} d^{-1} which is definitely counter balanced by the increase in nitrogen treatment efficiency.

SI.16 References

(1) Casagli, F.; Zuccaro, G.; Bernard, O.; Steyer, J.-P.; Ficara, E. ALBA: A Comprehensive Growth Model to Optimize Algae-Bacteria Wastewater Treatment in Raceway Ponds. *Water Res.* **2021**, *190*, 116734. https://doi.org/10.1016/j.watres.2020.116734.

(2) Dickson, A. G. The Development of the Alkalinity Concept in Marine Chemistry. *Mar. Chem.* **1992**, *40* (1–2), 49–63. https://doi.org/10.1016/0304-4203(92)90047-E.

(3) D. A. Wolf-Gladrow; Zeebe, R. E.; Klaas, C.; Körtzinger, A.; Dickson, A. G. Total Alkalinity: The Explicit Conservative Expression and Its Application to Biogeochemical Processes. *Mar. Chem.* **2007**, *106* (1–2), 287–300. https://doi.org/10.1016/j.marchem.2007.01.006.

(4) Henze, M.; Gujer, W.; Mino, T.; van Loosedrecht, M. Activated Sludge Models ASM1, ASM2, ASM2d and ASM3. *Water Intell. Online* **2000**, *5* (0), 9781780402369–9781780402369. https://doi.org/10.2166/9781780402369.

(5) Reichert, P.; Borchardt, D.; Henze, M.; Rauch, W.; Shanahan, P.; Somlyódy, L.; Vanrolleghem, P. E. River Water Quality Model No. 1. *IWA Sci. Tech. Rep. No 12* **2002**.

(6) Arashiro, L. T.; Rada-Ariza, A. M.; Wang, M.; van der Steen, P.; Ergas, S. J. Modelling Shortcut Nitrogen Removal from Wastewater Using an Algal–Bacterial Consortium. *Water Sci. Technol.* **2017**, *75* (4), 782–792. https://doi.org/10.2166/wst.2016.561.

(7) Batstone, D. J.; Keller, J.; Angelidaki, I.; Kalyuzhnyi, S. V.; Pavlostathis, S. G.; Rozzi, A.; Sanders, W. T. M.; Siegrist, H.; Vavilin, V. A. The IWA Anaerobic Digestion Model No 1 (ADM1). *Water Sci. Technol.* **2002**, *45* (10), 65–73. https://doi.org/10.2166/wst.2002.0292.

(8) Solimeno, A.; Parker, L.; Lundquist, T.; García, J. Integral Microalgae-Bacteria Model (BIO_ALGAE): Application to Wastewater High Rate Algal Ponds. *Sci. Total Environ.* **2017**, *601–602*, 646–657. https://doi.org/10.1016/j.scitotenv.2017.05.215.

(9) Decostere, B.; De Craene, J.; Van Hoey, S.; Vervaeren, H.; Nopens, I.; Van Hulle, S. W. H. Validation of a Microalgal Growth Model Accounting with Inorganic Carbon and Nutrient Kinetics for Wastewater Treatment. *Chem. Eng. J.* **2016**, *285*, 189–197. https://doi.org/10.1016/j.cej.2015.09.111.

(10) Jubany Güell, I. *Operation, modeling and automatic control of complete and partial nitrification of highly concentrated ammonium wastewater*; Universitat Autònoma de Barcelona, 2007.

(11) Wiesmann, U. Biological Nitrogen Removal from Wastewater. In *Biotechnics/Wastewater*; Advances in Biochemical Engineering/Biotechnology; Springer: Berlin, Heidelberg, 1994; pp 113–154. https://doi.org/10.1007/BFb0008736.

(12) M.K. de Kreuk; Picioreanu, C.; Hosseini, M.; Xavier, J. B.; van Loosdrecht, M. C. M. Kinetic Model of a Granular Sludge SBR: Influences on Nutrient Removal. *Biotechnol. Bioeng.* **2007**, *97* (4), 801–815. https://doi.org/10.1002/bit.21196.

(13) Martínez, C.; Mairet, F.; Bernard, O. Theory of Turbid Microalgae Cultures. *J. Theor. Biol.* **2018**, *456*, 190–200. https://doi.org/10.1016/j.jtbi.2018.07.016.

(14) Ginot, V.; Hervé, J.-C. Estimating the Parameters of Dissolved Oxygen Dynamics in Shallow Ponds. *Ecol. Model.* **1994**, *73* (3), 169–187. https://doi.org/10.1016/0304-3800(94)90061-2.

(15) Metcalf; Eddy. *Wastewater Engineering: Treatment and Resource Recovery*; McGraw-Hill education, 2014.

(16) Sander, R. Compilation of Henry's Law Constants (Version 4.0) for Water as Solvent. *Atmospheric Chem. Phys.* **2015**, *15* (8), 4399–4981. https://doi.org/10.5194/acp-15-4399-2015.

(17) Perry, R.H. *Perry's Chemical Engineers' Handbook, 9th Edition*; Mcgraw hill education, 2019.

(18) ASCE. *Measurement of Oxygen Transfer in Clean Water | Standards*; 1992.

(19) Barceló-Villalobos, M.; Guzmán Sánchez, J. L.; Martín Cara, I.; Sánchez Molina, J. A.; Acién Fernández, F. G. Analysis of Mass Transfer Capacity in Raceway Reactors. *Algal Res.* **2018**, *35*, 91–97. https://doi.org/10.1016/j.algal.2018.08.017.

(20) Ejiofor, A. O.; Posten, C. H.; Solomon, B. O.; Deckwer, W.-D. A Robust Fed-Batch Feeding Strategy for Optimal Parameter Estimation for Baker's Yeast Production. *Bioprocess Eng.* **1994**, *11* (4), 135–144. https://doi.org/10.1007/BF00518735.

(21) Vatcheva, I.; de Jong, H.; Bernard, O.; Mars, N. J. I. Experiment Selection for the Discrimination of Semi-Quantitative Models of Dynamical Systems. *Artif. Intell.* **2006**, *170* (4–5), 472–506. https://doi.org/10.1016/j.artint.2005.11.001.

(22) Solimeno, A.; Gómez-Serrano, C.; Acién, F. G. BIO_ALGAE 2: Improved Model of Microalgae and Bacteria Consortia for Wastewater Treatment. *Environ. Sci. Pollut. Res.* **2019**, *26* (25), 25855–25868. https://doi.org/10.1007/s11356-019-05824-5.

(23) Bernard, O.; Rémond, B. Validation of a Simple Model Accounting for Light and Temperature Effect on Microalgal Growth. *Bioresour. Technol.* **2012**, *123*, 520–527. https://doi.org/10.1016/j.biortech.2012.07.022.

(24) Rosso, L.; Lobry, J. R.; Flandrois, J. P. An Unexpected Correlation between Cardinal Temperatures of Microbial Growth Highlighted by a New Model. *J. Theor. Biol.* **1993**, *162* (4), 447–463.
(25) Ippoliti, D.; Gómez, C.; del Mar Morales-Amaral, M.; Pistocchi, R.; Fernández-Sevilla, J. M.; Acién, F. G. Modeling of Photosynthesis and Respiration Rate for Isochrysis Galbana (T-Iso) and Its Influence on the Production of This Strain. *Bioresour. Technol.* **2016**, *203*, 71–79. https://doi.org/10.1016/j.biortech.2015.12.050.
(26) Rosso, L.; Lobry, J. R.; Bajard, S.; Flandrois, J. P. Convenient Model To Describe the Combined Effects of Temperature and PH on Microbial Growth. *Appl. Environ. Microbiol.* **1995**, *61* (2), 610–616. https://doi.org/10.1128/AEM.61.2.610-616.1995.
(27) Lin, Y.; Guo, M.; Shah, N.; Stuckey, D. C. Economic and Environmental Evaluation of Nitrogen Removal and Recovery Methods from Wastewater. *Bioresour. Technol.* **2016**, *215*, 227–238. https://doi.org/10.1016/j.biortech.2016.03.064.
